# Fundamentals and Applications of Polymer Brushes in Air

**DOI:** 10.1021/acsapm.1c01615

**Published:** 2022-01-14

**Authors:** Guido C. Ritsema van Eck, Leonardo Chiappisi, Sissi de Beer

**Affiliations:** † Sustainable Polymer Chemistry Group, Department of Molecules & Materials, MESA+ Institute for Nanotechnology, University of Twente, P.O. Box 217, 7500 AE Enschede, The Netherlands; ‡ Institut Max von Laue - Paul Langevin, 71 avenue des Martyrs, 38042 Grenoble, France

**Keywords:** polymer brush, vapor, sensing, separations, wetting, adhesion, surfaces

## Abstract

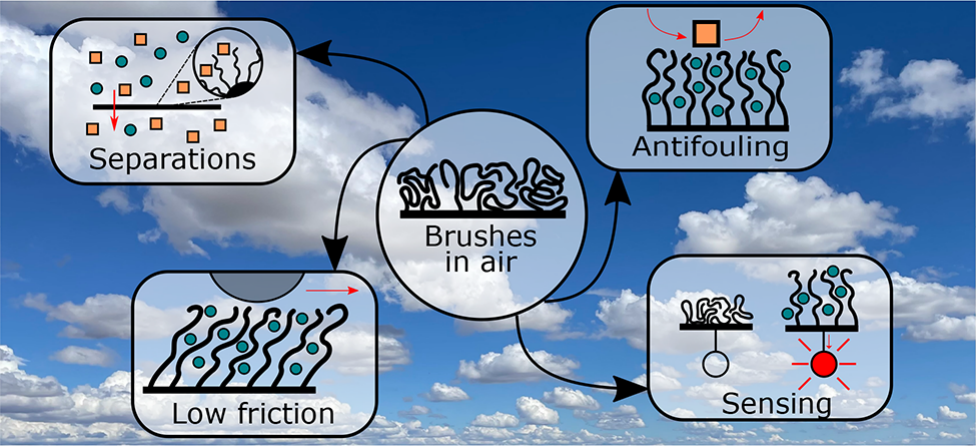

For several decades, high-density, end-tethered polymers, forming so-called polymer brushes, have inspired scientists to understand their properties and to translate them to applications. While earlier research focused on polymer brushes in liquids, it was recently recognized that these brushes can find application in air as well. In this review, we report on recent progress in unraveling fundamental concepts of brushes in air, such as their vapor-swelling and solvent partitioning. Moreover, we provide an overview of the plethora of applications in air (e.g., in sensing, separations or smart adhesives) where brushes can be key components. To conclude, we provide an outlook by identifying open questions and issues that, when solved, will pave the way for the large scale application of brushes in air.

## 1 Introduction

Polymer brushes are composed of long macromolecules that are anchored by one chain-end to a surface at a density that is high enough such that the polymers stretch out, away from the surface.^1^ These brushes have become popular surface modifications^2^ in the development of bioinspired lubricants^3^ and/or antifouling^4−6^ and antimicrobial surfaces.^7,8^ As such, they can be broadly applied, ranging from (bio)medical materials^9^ to membrane technologies.^10,11^ Moreover, polymers are responsive to small changes in their environment, such as temperature, pH, or solvent composition.^12,13^ Consequently, the properties of polymer brushes alter in response to their environment as well, which has been utilized to control adhesion and friction,^14,15^ channel flow,^16,17^ drug release,^18,19^ and more.^20^

In recent years, the interaction between polymer brushes and gaseous media has become a subject of research attention. Brushes may swell, similar to their liquid-solvated state, by capturing vapors of a favorable solvent. As a result, polymer brush functionalization can provide selectivity and enhanced responsiveness in various gas sensor designs,^21,22^ induce the formation of long-ranged superstructures by vapor-annealing brush coated particles^23^ or enable the formation of perfectly smooth metal coatings.^24^ The same sorption behavior has also been combined with thermoresponsive polymers to create materials with temperature-dependent water sorption, with applications in moisture capture and management.^25,26^ Moreover, previously outlined applications such as antifouling^27^ and lubrication^28−30^ may also be extended to surfaces in air. However, polymer brushes in air deviate qualitatively from their behavior in liquids, both at interfaces^31^ and in the bulk.^32^ This has given rise to new scientific questions that need answering to enable and optimize the application polymer brushes in air.

In this work, we aim to provide an overview of the fundamental considerations that are relevant to polymer brush research in air, and of the steps taken toward particular applications in the last two decades. We emphasize generally relevant physical phenomena and chemical effects that are exemplary of broad classes of materials. In section 2, we introduce the basic concepts of polymer brush physics, and then, we discuss how vapor absorption isotherms and vertical density profiles arise from the structure of the polymer brush. Furthermore, we qualitatively discuss the more complex mixing effects and phase separations that may occur in the case of vapor mixtures and mixed brushes. In section 3, we cover developments in polymer brush research specific to the fields of gas sensing, membrane separations, control of friction and adhesion, and wetting. Here, we include both experimental steps toward these applications and fundamental work of particular relevance to these applications. Finally, we discuss important open questions and issues for the large-scale applicability of brush-based technologies in section 4.

## 2 Fundamentals

### 2.1 Introduction to Polymer Brushes (in Air)

In this section, we explain the basic properties and synthesis of polymer brushes. We describe the distinctive height scaling and solvation response of the brush, explain the main contributions to the free energy, and we outline commonly used thermodynamic descriptions of the brush, in particular for brushes in air.

A polymer brush is a coating comprised of polymer chains, end-anchored to a substrate at a high areal density. These brushes can be composed of negatively charged anionic or positively charged cationic polyelectrolytes,^33,34^ zwitterionic polymers,^35^ and neutral macromolecules or copolymers containing different types of monomers.^36^ Individually, surface-anchored polymers behave comparably to free polymers, assuming conformations that minimize their free energy, which consists of contributions from solvent, substrate, and polymer–polymer contacts, and the conformational entropy of the chain. In the simplest case, this is a “mushroom”: a surface-anchored analogue to the coil and globule states found in free polymers. Under poor solvent conditions, however, the most favorable conformation is often a ”pancake” state in which the polymer backbone adsorbs to the grafting surface.^37^ When the density of polymers on the surface becomes sufficiently high, the polymers start to overlap and volume interactions cause the chains to stretch away from the surface. This structure of ”bristles” extending away from the substrate gives the polymer brush its name. The transition from individual chains to a brush is frequently described in terms of the reduced tethering density:^38^
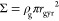
where ρ_g_ is the number of chains per unit area and *r*_gyr_ is the radius of gyration of a single grafted chain under the given conditions of solvent and temperature. Hence, Σ represents the number of chains that occupy the surface area covered by a single chain under ideal conditions. While Σ < 1 leads to nonoverlapping mushrooms or pancakes by this definition, Σ > 1 does not necessarily imply a highly extended polymer brush. The point at which a grafted polymer layer starts to display the characteristic scalings of a brush appears to differ from system to system, but generally occurs for Σ > 5.^38,39^ An interesting intermediate regime occurs for brushes in the approximate 1 < Σ < 5 range under poor solvent conditions. In this situation the brush may separate into inhomogeneous aggregates on the substrate, sometimes called ”octopus micelles”,^40^ which minimize the free energy of unfavorable solvent interactions at the expense of chain stretching. Curiously, these octopus micelles can only be formed when the grafted polymers collapse rapidly; for a slow decrease in solvent quality, polymers will individually collapse into mushrooms, which can no longer aggregate once the solvent quality has decreased sufficiently to make this favorable.^41^

**Figure 1:**
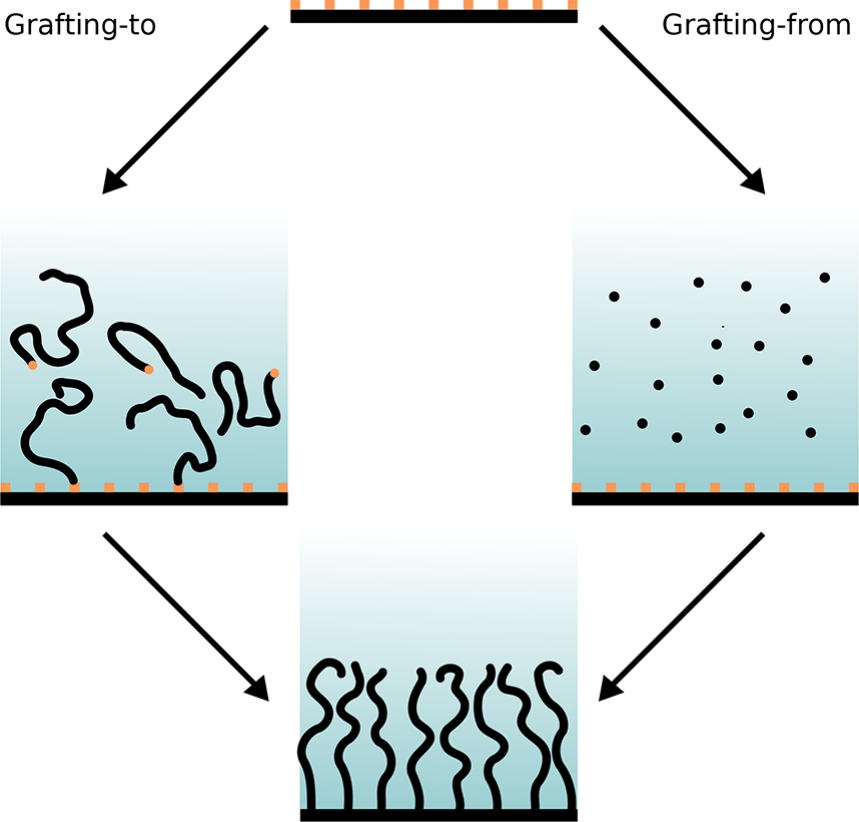
Schematic illustration of the two broad strategies for polymer brush synthesis: in grafting-to, polymer chains with reactive end groups are attached to anchoring groups (both represented in orange) at a surface from solution, whereas in grafting-from, polymerization occurs in situ from surface-bound initiators.

The methods of synthesizing polymer brushes fall into two broad categories, illustrated in Figure 1: grafting-from and grafting-to approaches. In grafting-from procedures, the substrate is functionalized with initiator moieties and the brush is grown by in situ polymerization, whereas in grafting-to, fully grown polymer chains are chemically or physically attached to the substrate.^42^ Grafting-from typically employs controlled radical polymerization methods (e.g., atom transfer radical polymerization, reversible addition–fragmentation chain-transfer, or nitroxide-mediated polymerization) or ring-opening metathesis where applicable.^43,44^ While this restricts the range of available monomers and synthetic conditions, grafting-from strategies nonetheless remain highly relevant due to their ability to produce brushes with a high areal density of chains. On the other hand, the use of prefabricated polymers in grafting-to affords much greater control of the architecture, molecular weight and dispersity of the polymer, but steric interactions between chains strongly limit the attainable grafting density.

**Figure 2:**
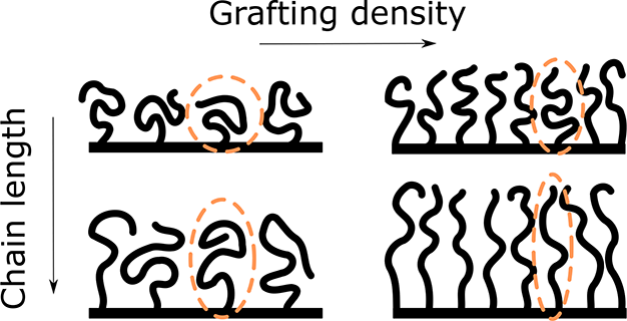
Illustration of the effects of grafting density and chain length on polymer brush behavior. In low-density coatings of short polymers, each chain individually takes on an energy-minimizing conformation. As the chain length and grafting density increase, excluded volume interactions cause the chains to extend.

Much like free polymers in solution, polymer brushes are highly sensitive to the conditions of the surrounding medium.^45−47^ Under poor solvent conditions, neutral polymers are effectively attracted to one another at medium distances and the brush collapses into a dense layer, limited only by short-range repulsions (e.g., Pauli exclusion). For miscible polymer–solvent combinations, however, the net interaction between polymer segments becomes repulsive. In this case, the equilibrium brush height is determined primarily by a balance of interactions between chain segments and the entropic elasticity of the chains. As a result, the height of a polymer brush depends on both the length of the polymer chains and the density at which they are anchored to the surface, as visualized in Figure 2. Minimizing the free energy as a function of height for the true brush regime (Σ > 5) yields *h* ∼ ρ_g_^1/3^*N*, where *h* represents the brush height and *N* the chain length. This result has long been noted as significant,^48^ since the linear relation between height and chain length implies that the polymer chains are strongly stretched. This marks a clear deviation from the free polymer behavior, where the radius of gyration of the polymer coil is isotropic and scales with a solvent-dependent Flory exponent. The scaling relation for the brush height has been experimentally validated.^49−51^ Yet, at very high densities, the exponent for the dependence on ρ_g_ increases further, as three-particle and higher order interaction parameters start to impact the free energy.^52,53^

Polymer brushes under gaseous atmospheres tend to collapse as in poor solvents, since the low density of gases presents them with very few energetically favorable interactions. However, interesting behaviors arise when polymer brushes in air are exposed to good solvents. Solvent droplets placed on brushes may spread or partially evaporate, and conversely solvent vapors may condense in the presence of the brush. The composition of a solvated brush in air therefore depends on the concentration of the solvent vapor. To our knowledge, the first work to describe the swelling of polymer brushes by vapors was presented over two decades ago by Brochard-Wyart and De Gennes, theoretically investigating the capillary rise of a liquid against a brush-covered plate.^54^ In experimental work, brush swelling by vapors is often characterized in terms of an effective interaction parameter, by imposing chemical equilibrium between the vapor and a Flory–Huggins (FH) type description of the brush.^55−59^

Notably, some of these works^55,59^ follow Birshtein and Lyatskaya^60^ in modifying the FH model to account for the entropic elasticity of the polymer chains. In typical cases, the elastic contribution is small relative to enthalpic terms, as is reflected in experiments where brush swelling is independent of grafting density.^58,61^ However, it is important to include an elasticity term when describing strongly absorbing brushes in vapors near saturation, as it enforces a finite brush height, differentiating the brush from a free polymer melt in the FH description. We discuss the quantitative details of these models in section 2.2.

### 2.2 Isotherms

Vapor sorption is commonly described in terms of a sorption isotherm: the relation between a vapor’s pressure relative to saturation (for water: the relative humidity) and the ab- or adsorption of this vapor. This is highly relevant from both theoretical and experimental perspectives, since the pressure of a vapor is an important thermodynamic quantity, as well as a typical experimental variable. In theoretical and simulation works, absorption is generally represented by the solvent volume fraction ϕ_s_, whereas in experimental settings the ratio between the swollen height and the dry height of the brush, known as the swelling ratio , is commonly used due to its ease of measurement. Assuming that the brush does not contain voids and the volume of the polymer and solvent does not change upon mixing, these quantities are related by  In this section, we quantitatively discuss the absorption isotherms predicted by a modified Flory–Huggins model, and compare with experimentally obtained isotherms.

The shape of the absorption isotherm is dictated by the chemical equilibrium between a bulk vapor reservoir and the solvent sorbed in the brush. Typically, the chemical potential of the bulk solvent vapor phase is assumed to be constant; experimentally, this corresponds to a large reservoir or atmosphere of solvent vapor. Since chemical equilibrium requires the absence of chemical potential gradients, this means the absorption isotherm is determined by the free energy of the solvated brush, of which the derivative w.r.t. the number of absorbed solvent particles provides the chemical potential of sorbed solvent. In the extended Flory–Huggins theory by Birshtein and Lyatskaya^60^ discussed in section 2.1, the free energy of the brush is given by
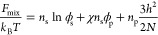
1where *n* and ϕ are the number of particles and the volume fraction of a species, with subscripts s and p denoting solvent and polymer respectively, χ is the Flory–Huggins interaction parameter for polymer–solvent contacts, and *N* is the degree of polymerization for all chains. This expression deviates from the standard Flory–Huggins model in the omission of a translational entropy term for polymer chains, and the addition of the last addend, which approximates the entropic cost of brush swelling. Classically, describing a three-component system of polymer, solvent and air would require interaction parameters for each of the three two-component pairs in the system. However, interactions including air or vacuum can be neglected under the assumption that the fraction of void sites in the brush is very small.

The chemical equilibrium condition for this modified model is stated as
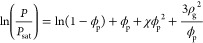
2with *P* the partial pressure of the vapor, *P*_sat_ the saturation pressure of the vapor, ϕ_p_ the volume fraction of polymer in the brush, and χ the Flory–Huggins interaction parameter between solvent and polymer. The left-hand side of eq 2 represents the chemical potential of the solvent vapor, assuming ideal gas conditions. The right-hand side, which is the derivative of eq 1 with respect to the amount of absorbed solvent, is the chemical potential of solvent in the brush. This defines the absorption isotherm for fixed χ and ρ_g_. Further discussion of this model can be found in refs (60 and 62). While this description was originally proposed for a brush in a one-component solvent (where the left-hand side of eq 2 is always zero), it is equally applicable to gaseous environments, requiring only the assumption that the brush is free of voids. Moreover, this gas-phase scenario can also be compared to a solute or minority component in a background medium that is unfavorable to both the solute and the polymer, although this requires the additional assumption that the background medium interacts equally with the solute and the polymer. This extension to the gas phase does disregard Schroeder’s paradox, however. While a liquid and a saturated vapor should solvate the brush identically from a thermodynamic perspective, liquids have been experimentally found to swell polymer materials considerably more than saturated vapors. This result, known as Schroeder’s paradox, was found for gelatin by Schroeder in 1903, and it has recently attracted renewed attention for its relevance to polymeric membrane materials.^63,64^ However, as no conclusive thermodynamic explanation for this effect exists as of yet, this cannot be accounted for theoretically.

**Figure 3:**
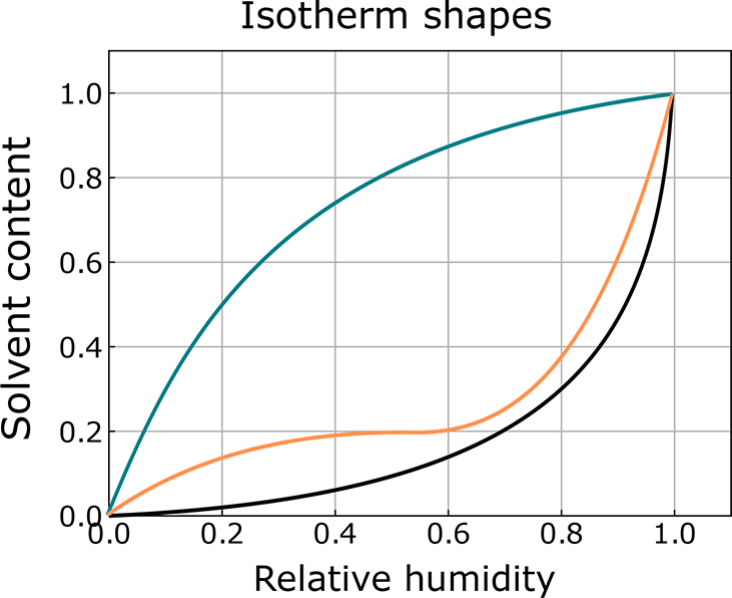
Approximate shapes of the different types of absorption isotherms discussed in this section. Black: Typical concave-upward Flory–Huggins isotherm. Blue: Concave-downward Flory–Huggins isotherm, found in extremely strongly interacting systems. Orange: Isotherm with crossover from void-filling regime to Flory–Huggins behavior at higher relative humidities. The vertical axis is normalized by the solvent content at saturation for the given system. The curves shown here are strictly illustrative and should not be used for any quantitative comparison.

The isotherms described by eq 2 take on a concave-upward shape for positive or weakly negative values of χ, whereas strong attractive interactions (large negative χ) shift the isotherm toward a concave-downward shape. In most experimental systems, the concave-upward shape, shown in black in Figure 3, is observed,^56−58,61,65,66^ in line with the generally positive χ parameters of real solvent–polymer systems. Although less frequently reported, the concave-downward isotherm, shown in blue in Figure 3 is attainable in polyelectrolytic or densely hydrogen-bonding systems in water.^58,67^ The concave-upward case is similar to the type 3 isotherm in the Brunauer–Deming–Deming–Teller (BDDT) classification,^68^ in which the vapor’s energy of condensation drives adsorption onto a weakly attractive substrate: for positive FH parameters, brush-solvent contacts are enthalpically unfavorable, but absorption is driven by the entropy of vapor molecules entering the volume of the brush. The enthalpic cost per vapor molecule only decreases as the solvent fraction increases, leading to the upward curvature of the isotherm. The concave-downward form of the isotherm loosely resembles the Langmuir (BDDT type 1) isotherm in the sense that sorption is enthalpically driven and limited by the sorption capacity of the substrate, but these limits are different in origin.

Experimental isotherms may display additional features when this lattice model does not fully capture the free energy of the system, however. For instance, the assumption that brush swelling is linearly related to solvent uptake does not always apply to real systems, since polymeric materials often contain free volume that may be filled by solvent.^65,69^ This effect is most pronounced in materials below their glass transition temperature^65^ and in ”stiff” polymers, i.e., polymers with large persistence lengths.^70^ Absorption by void-filling leads to increased solvent uptake at low pressures when compared to the Flory–Huggins-based isotherm, and Laschitsch et al. suggest that the transition from void-filling to a Flory–Huggins regime may be associated with a solvent-induced glass transition.^65^ Single-stranded DNA (ssDNA) brushes in water vapor may even collapse with increasing relative humidity. This effect was first described by Wagman et al., in terms of a lattice model incorporating void sites, and later by Zhao et al., based on hydrogen bonding between sorbed water and the ssDNA strands.^32,71^

**Figure 4:**
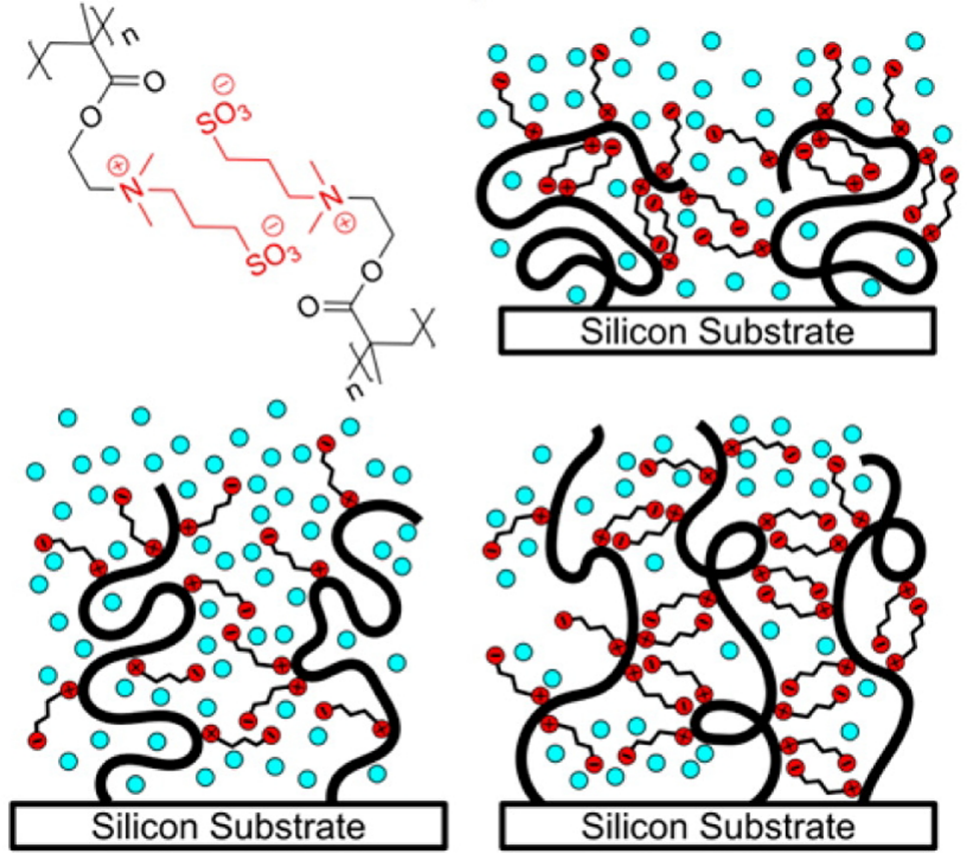
Mechanism proposed for nonmonotonous swelling of polyzwitterionic brushes by Galvin et al.^57^ Top left: Structure of the sulfobetaine side chain complex. Top right: intramolecular association of ionic groups in low-density brushes. Bottom left: minimal association of ionic groups in brushes of moderate density. Bottom right: intermolecular association of ionic groups in dense brushes. Reproduced with permission from ref (57). Copyright 2014 American Chemical Society.

A related effect was observed by Galvin et al. in a neutron reflectivity (NR) study of polyelectrolyte brushes:^57^ a brush containing zwitterionic sulfobetaine side groups displayed different sorption behavior in humid air depending on its grafting density, with Flory–Huggins type behavior at moderate grafting densities but unexpectedly low solvent uptake at both low and high grafting densities (see Figure 4). This was attributed to the collapse of the brush by formation of side group complexes within chains at low grafting densities (top right Figure 4) and between chains at high densities (bottom right Figure 4), with the intermediate systems being unable to form a high density of either type of complex (bottom left Figure 4). Moreover, these high-density zwitterionic brushes appeared to swell while maintaining or decreasing their solvent content under some conditions. This suggests a long-range restructuring, in which the formation of interchain complexes also increases the free volume within the brush.

Löhmann et al. report that isotherms for polyelectrolyte brush/polyelectrolyte multilayer composites in water vapor can be shifted by modifying the thickness of the multilayer component.^72^ Moreover, at high relative humidities, a water-enriched region forms in between the brush and multilayer components. Although these isotherms are reported in terms of the swelling ratio, excluding void-filling effects, they display a regime transition that superficially resembles the one seen in ref (65). The shift from swelling of the individual components at low relative humidity to accumulation of solvent in the intermediate region at high relative humidity appears to be the cause of this two-regime isotherm.

#### 2.2.1 Outlook on Isotherm Modeling

Since the shape of the isotherm follows from a chemical equilibrium condition, improving upon the model discussed here primarily requires a more exact free energy description for the brush. Accounting for the distribution of chain end positions and differences in composition over the brush height may quantitatively improve on eq 2, but will typically not alter the scaling.^73^ However, various further adjustments may improve the results for specific systems. In the case of hydrogen bonding or complexation between polymer and solvent, a composition-dependent expression for χ could be used to account for saturation of the relevant functional groups. Nevertheless, the most broadly relevant open issue for brushes in air specifically is the effect of free volume. While the energetic effect of free volume can be reasonably well described within a lattice model, we are not aware of a predictive theory relating the free volume fraction in a brush to the solvation state of the brush and the persistence length of the polymer. This would close the main discrepancy in knowledge between vapor-solvated and liquid-solvated brushes, and likely provide valuable insight into glass transitions in polymer brushes. Finally, sorption isotherms are an indication of equilibrium behavior only. In practical applications, the response time of the brush to a change in solvent composition may be important as well. Therefore, systematic exploration of the kinetics of brush swelling in different brush/vapor systems may be important in translating brush thermodynamics to design parameters.

### 2.3 Density Profiles

The absorption behavior of polymer brushes can largely be described by bulk models, in which the overall composition of the brush is considered. However, solvent and polymer are not always evenly distributed throughout the brush, as the effect of chain stretching depends on the distance from the grafting plane. This variation in composition influences the interfacial properties of the polymer brush, making it highly relevant for surface functionalizations. In this section, we provide an overview of the literature that describes the density profiles for solvated polymer brushes. Next, we show how brushes in vapor may deviate from these profiles due to interfacial effects and incomplete solvation.

**Figure 5:**
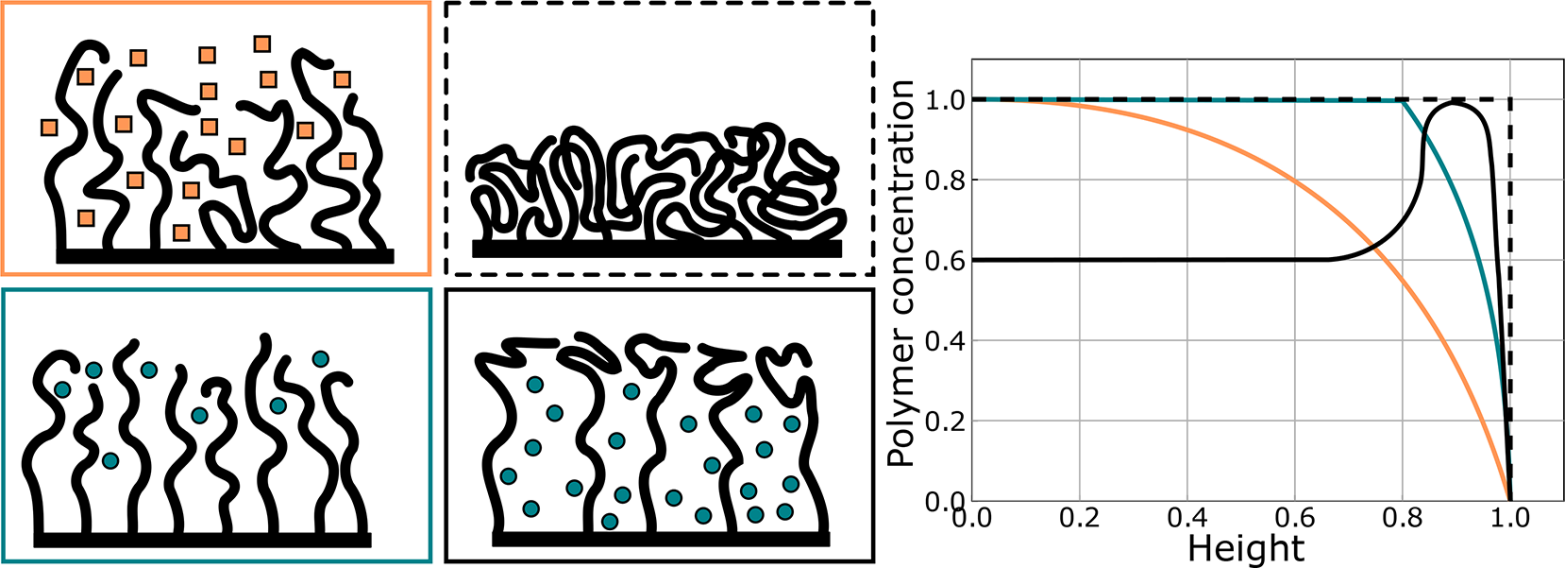
Polymer concentration against distance from the grafting plane, qualitatively represented for a number of the scenarios described in this section. Axes are normalized against the brush height and maximum polymer density for any given system, meaning that these should not be quantitatively compared. Orange: the density in a fully solvated neutral brush decays parabolically away from the grafting surface. Dashed: Dry and poorly solvated polymer brushes are completely collapsed, leading to a step-like density profile. Neglecting interfacial effects, this profile is also expected for nongrafted polymer films. Blue: Polymer brushes undersaturated with a good solvent maintain a constant density near the surface, but decay parabolically near the brush-air interface.^82^ This is comparable to vapor-solvated systems below the saturation pressure. Black: the theoretical floating brush scenario, in which chains in an undersaturated polymer brush adsorb at the liquid–vapor interface.

The physical properties of a solvated polymer brush depend not only on its composition, but also on the conformation of chains within the brush. In the preceding sections, we discussed mostly “box-like” descriptions of the brush, in which all chains are extended to the full brush height and the composition of the brush is homogeneous over its height in both good and poor solvents. Although such models are convenient and adequately predict some of the relevant scalings,^39,50,74^ the assumption that all chains ends are located at the same distance from the substrate is unrealistic from an entropic perspective. In reality, chain conformations within a single brush may range from dense states close to the grafting plane to highly extended chains reaching all the way to the outer edge of the brush. Brushes at high grafting densities may still display step-like density profiles, as observed in both experiments^75^ and simulations,^62,76^ since chains are sufficiently extended to describe the elastic contributions to the free energy by a mean-field argument.^73^ Under moderate conditions, however, a more complex profile arises. Several self-consistent field studies have shown that the polymer concentration in a brush decays parabolically away from the grafting plane,^1,73,77^ i.e., ϕ_p_(*z*) ∼ *C* – *z*^2^, where *C* is a constant depending on the brush parameters and *z* the distance from the grafting plane. An approximation of this density profile is shown in orange in Figure 5. Additionally, in real systems, unconstrained chain ends near the outer edge of the brush create a short, Gaussian “tail” region far from the grafting surface.^78^ Interestingly, this parabolic model retains the same height scaling of *h* ∼ ρ_g_^1/3^*N* as its box-like counterpart. Brushes in poor solvent retain their step-like density profile (dashed in Figure 5) in this description, since enthalpic and entropic contributions both favor the collapse of the polymer in this case. Interestingly, phase segregation is predicted to occur for polymers with a concentration dependent effective interaction (such as poly(*N*-isopropylacrylamide) (PNIPAm)), at the transition between the parabolic and box-like phases.^79^ This will result in a high density collapsed phase near the substrate and a low density swollen phase on top of it.^80^ Yet, it has been difficult to confirm this experimentally.

The density profiles of polymer brushes in air may differ from ideal liquid-solvated brushes in many ways, however. For instance, the presence of a liquid–vapor interface may give rise to various boundary effects. When a boundary between a condensed phase and a gas or vacuum exists, molecules in the region near this boundary experience fewer intermolecular interactions than molecules in the bulk. In mixtures, this causes the species that experiences the weakest average interaction to migrate toward the surface, minimizing the energetic cost of the interface. Since interactions between two different species are symmetric, this is typically the component with the weaker self-interaction, i.e., the lower surface tension. Indeed, Sun et al. found results consistent with solvent enrichment at the liquid–air interface for neutron reflectivity (NR) measurements of polystyrene brushes in toluene vapor.^31^ Furthermore, their experimental results and self-consistent field theory indicate that the polymer density profiles of these brushes are similar to the expected parabolic profiles for brushes in liquid, mainly deviating in the fact that the polymer density abruptly drops to zero near the free interface rather than decaying gradually. Another NR study on brushes of the weak polyelectrolyte poly(2-(dimethylamino)ethyl methacrylate) (PDMAEMA) in humid air by Galvin et al. showed similarly enhanced water concentrations near the air interface.^57^ Dissipative particle dynamics simulations^81^ and molecular dynamics simulations^62^ of chemically nonspecific polymers also display this adsorption, indicating that it does not depend on any specific chemical effect.

Adsorption phenomena are not restricted to the solvent, however; many water-soluble polymers are surface-active at water–air interfaces due to their low surface tension relative to water.^83^ Self-consistent field studies suggest that the free ends of grafted chains could similarly adsorb to the water–air interface, and this in fact influences the wetting behavior of brushes.^84^ However, to our knowledge, the existence of a ”floating brush” (approximated by the black line in Figure 5), where a brush anchored to a solid substrate displays an enrichment in polymer at the liquid–air interface, has never been reported in experiment or simulation. Even in spin-coated polymer films, where polymer migration to the surface is not hindered by grafting, solvent-enriched layers have been observed at the polymer–air interface in experiment.^85,86^

Finally, a good solvent vapor at low pressures will not necessarily condense in sufficient amounts to fully solvate the brush, resulting in partially swollen states. Goedel et al. expanded upon the analyses that yielded the parabolic brush profile to show that the density profiles of such intermediate states are truncated parabolas (shown in blue in Figure 5); that is, they display a constant polymer density near the grafting plane, but decay parabolically at large distances.^82^ While this work does not explicitly consider vapors, the situation of a partially swollen brush appears thermodynamically similar to vapor solvation.

#### 2.3.1 Outlook on Density Profile Characterization

Density profiles under liquid-solvated conditions have been thoroughly researched for neutral and charged polymer brushes in a variety of regimes. The principles that give rise to these profiles also apply to polymer brushes in air, suggesting that these results are relevant here as well. However, the effect of the brush–air interface is comparatively unexplored. The width of the interface is of particular interest, as this would inform calculations of the surface energy. Additionally, as Sun et al. point out, the shape of the brush-air interface influences surface fluctuations.^75^ Another open question is the existence of the ”floating brush” state described earlier in this section. Beyond theoretical curiosity, the floating brush state could lead to more thermally stable solvent binding, as the polymer-enriched layer would present a physical barrier to evaporation. Moreover, as a mechanically stable coating of surface-active polymer chains, the floating brush is likely to possess interesting wetting properties.

### 2.4 Vapor Mixtures

Polymer brushes’ responsive nature and ability to capture solvent molecules leads to diverse swelling behaviors in mixed solvent environments. We provide an overview of relevant research on polymer coatings in mixed vapors, and discuss the phenomena of cosolvency and cononsolvency and their applicability to gaseous systems.

**Figure 6:**
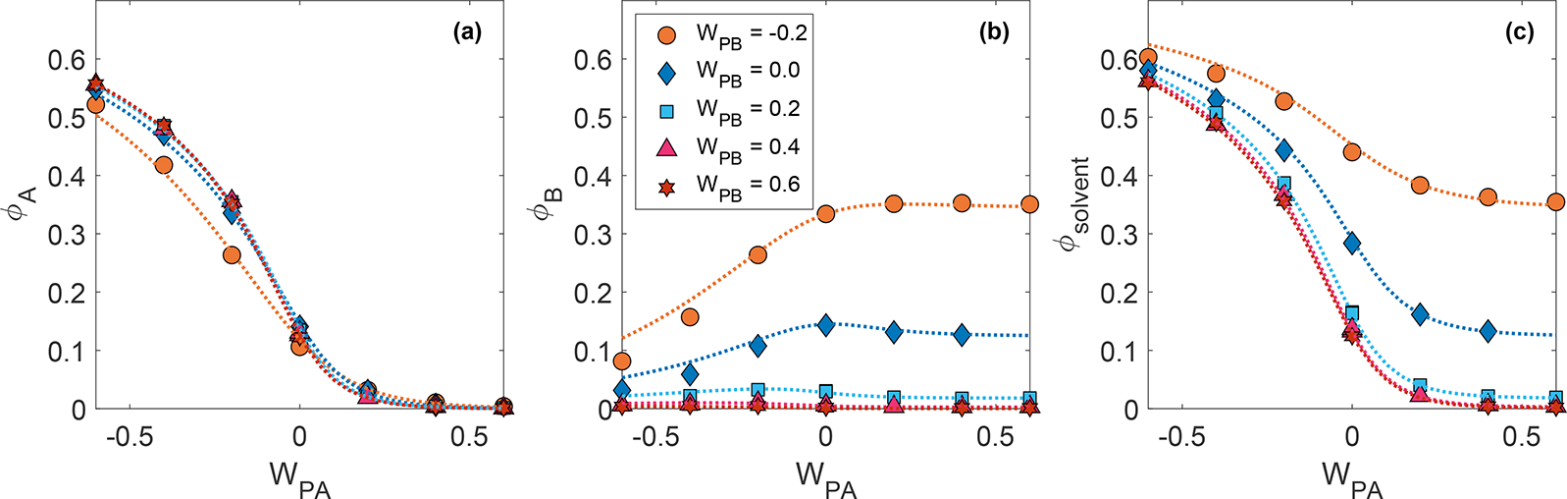
Composition of the polymer brush-vapor system described in ref (87), consisting of a polymer brush in contact with a 50/50 mixture of two solvents. (a) Volume fraction of solvent A in the brush, (b) volume fraction of solvent B in the brush, and (c) total solvent fraction in the brush as a function of the polymer-A interchange energy *W*_PA_ for a range of *W*_PB_ values. Markers indicate simulation results, dashed curves are theoretical predictions based on a ternary Flory–Huggins-like model. Reproduced with permission from ref (87). Copyright 2020 American Chemical Society.

When a polymer brush is exposed to vapor mixtures, multiple effects can occur depending on the relative affinity of the vapor with the brush. Already for binary vapor mixtures, nontrivial swelling behavior can be observed. A coarse-grained molecular dynamics study by our group shows that, even in the absence of cononsolvation, a mixture of two good solvent vapors may produce a range of enthalpy-driven absorption behaviors depending on the vapor composition and energy of interaction between the brush and both solvents.^87^ We identify preferential sorption of the better solvent whenever the two vapors are chemically distinct. In highly swollen, nearly saturated brushes, this leads to competition between the two solvents; at this point, increasing the polymer affinity of the preferred solvent causes very little additional swelling, but rather, it leads to the displacement of the secondary solvent out of the brush. Finally, when the affinity between the two solvents is stronger than the affinity of the secondary solvent for the brush, collaborative absorption may occur, in which the initial absorption of the preferred solvent creates a more favorable environment for the absorption of the secondary solvent. Figure 6 shows the composition of a polymer brush in contact with a mixture of vapors under variation of the polymer–solvent interaction parameters, represented by the polymer–solvent interchange energy *W*_Pi_ = −ϵ_Pi_ + ^1^/_2_(ϵ_ii_ + ϵ_PP_), with ϵ representing the energy of a single binary interaction, subscript P denoting the polymer, and subscript i indicating the solvent species. This quantity differs from the Flory–Huggins parameter χ only by a factor of *zk*_B_*T*, with *z* the coordination number for particles in the solution. As a result, it is frequently convenient in describing molecular dynamics simulations, where the interaction energies are directly controllable, but the coordination number is not. Preferential absorption can be seen in the fact that A and B are not absorbed in equal measure when A and B are not chemically identical. Competitive absorption manifests as a decrease in B content at negative *W*_PA_ for the topmost two curves in 6b (meaning absorption of B decreases as the quality of the competing solvent is increased). Last, collaborative absorption is shown by the subtle increase in B fraction at negative *W*_PA_ for the curves with positive *W*_PB_ in 6b (indicating that, despite being a poor solvent, a small fraction of B is absorbed when A is a good solvent due to their miscibility).

**Figure 7:**
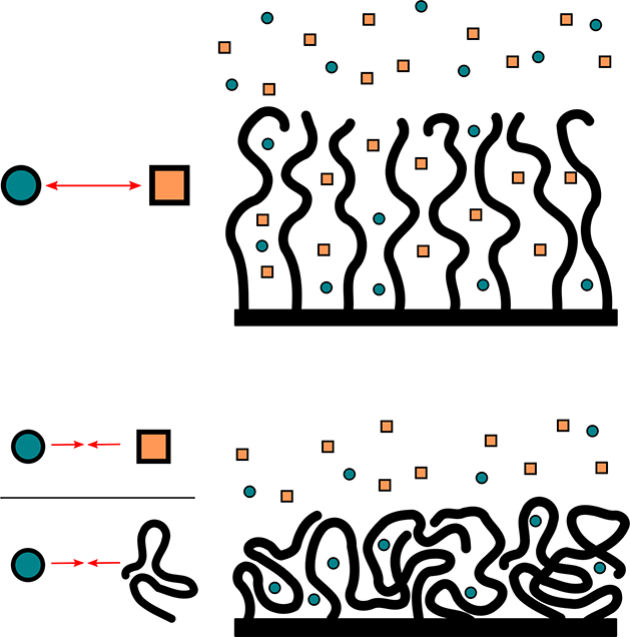
Schematic representation of cosolvation and cononsolvation in polymer brushes. Top: cosolvency is driven by repulsive interactions between miscible poor solvents, causing them to swell the polymer brush. Bottom: Cononsolvency causes the brush to collapse, usually containing a small amount of the minority solvent. While the exact mechanism remains to be clarified, it occurs mainly in systems with a strong preference of the polymer for one of the two solvents, or a strong interaction between the two solvents leading to avoidance of the polymer.

Cosolvency, illustrated in the top half of Figure 7, is a phenomenon in which a mixture of two poor solvents may, as a whole, form a good solvent for a polymer. Although this effect is counterintuitive, it can be explained with mean-field models.^88−90^ We refer the reader to the cited works for a comprehensive discussion of the thermodynamics, but we provide a qualitative argument. Approximating the solvent mixture as a single liquid yields an effective Flory–Huggins parameter χ_P,L_ = ϕ_*A*_χ_P,A_ + ϕ_*B*_χ_P,B_ – ϕ_*A*_ϕ_*B*_χ_A,B_, where subscripts P, L, A, and B indicate the polymer, solvent mixture, solvent A, and solvent B respectively, and the volume fractions ϕ apply to the composition of the solvent mixture. The key feature here is that a small solvent particle gains significant translational entropy upon mixing, while this gain is negligible for a large polymer coil. As a result, solvents with relatively high interaction energies may still be miscible (for χ_A,B_ < 2), whereas only a weakly repulsive interaction is required for the solvent to form a poor medium for the polymer (χ_P,A_ > 0.5 and χ_P,B_ > 0.5.) When these conditions are met, the individual solvents may interact with the polymer purely in order to minimize their contacts with each other, reducing their overall free energy. Cosolvency provides a way to switch the swelling state of a brush without fully replacing the solvent bulk, making it of interest for, e.g., switchable adhesion applications.^91^ Since cosolvency is driven by the enthalpy of exchanging solvent–cosolvent contacts for polymer–solvent and polymer–cosolvent contacts, it requires the presence of a solvent-rich phase for these solvent–cosolvent contacts to occur. As both solvents are individually poor, a polymer brush will not absorb a substantial fraction of solvent in a vapor environment, so solvent–cosolvent contacts are unlikely to be abundant within the brush. Poor solvents may still form a liquid adsorption layer on top of the brush when their surface tension is lower than that of the polymer, as discussed in section 2.3. However, this scenario is incompatible with the theory outlined in ref (88), which predicts full solubility of the polymer only when its cohesive energy density (a measure closely related to surface tension^92^) is intermediate between those of the two solvents. This suggests that substantial adsorption of both solvents is incompatible with cosolvency, and seems to preclude cosolvation by vapors. We do point out that the work of Scott assumes a Hildebrand description of miscibility, which is best suited for apolar materials. Hence, vapor cosolvation in highly polar or otherwise strongly interacting systems may still be possible.

Cononsolvency, counterpart to cosolvency, describes situations in which a mixture of two good solvents causes a polymer to collapse as if in a poor solvent. This scenario is shown in the bottom panel of Figure 7. PNIPAm–water–alcohol mixtures, which display an abrupt collapse and re-entrant swelling transition at low alcohol fractions, are the most well-known example of this phenomenon, and are often used as model systems.^93^ Unlike cosolvency, cononsolvency has not been conclusively explained, and the underlying mechanisms are a subject of active research. Some of the potential explanations focus on the interaction between the two solvents: Liu et al. propose that cononsolvency in the PNIPAm–water–alcohol system is caused by the composition-dependent formation of water–alcohol clusters, which have a weaker tendency to form hydrogen bonds than either of the pure solvents.^94^ The stoichiometry of these clusters may also explain the asymmetric relation between solvent composition and solvent quality. A lattice model by Dudowicz et al. shows that two highly miscible, moderately good solvents can produce cononsolvency by avoiding polymer–solvent contacts in favor of mixing between the two solvents.^89^ Although this model does not account for solvent clustering and covers a limited parameter space, it agrees with ref (94) in attributing cononsolvency primarily to solvent–solvent interactions. However, the majority of proposed mechanisms are centered around the polymer–solvent interactions. Tanaka et al. emphasize competition between solvents in forming hydrogen bonds with the polymer, and develop a model similar to competitive adsorption.^95^ Their theory indeed predicts a minimum of bonded solvent molecules per polymer at intermediate solvent compositions. Mukherji et al. also take an adsorption-based approach to the cononsolvency phenomenon but without relying on any specific chemical interactions. Instead, they propose that a minority fraction of the preferred solvent may cause multiple monomers to adsorb to each solvent particle, thereby causing the collapse of the polymer, whereas larger amounts of the preferred solvent would simply solvate the polymer chain in its entirety.^96^ This description was expanded to polymer brushes by Sommer,^97^ and further tested in molecular dynamics simulations.^98^ Rodríguez-Ropero et al. advance a model that is also based on preferential solvent binding, but they propose that methanol stabilizes collapsed conformations of the PNIPAm chain entropically rather than enthalpically through chemistry-specific effects.^99^ Later work in the same group suggests that preferential solvent binding is not required for cononsolvency, supporting the claim that the cononsolvency of the PNIPAm-water–alcohol system is chemistry-dependent.^100^ Recent research tends to view solvent mixing and preferential solvation both as causes of the cononsolvency phenomenon, rather than favoring one explanation over the other.^101^ This is supported by theoretical work by Zhang et al., which shows that both these phenomena can be described under the same random phase approximation.^102^

The apparent mechanisms of cononsolvency could, under suitable conditions, also apply to polymer brushes in gaseous environments. Mean-field solvent mixing, in which solvents prefer mixing over solvating the polymer, is the only scenario that seems unlikely, given the high polymer concentration in a brush and the absence of a bulk solvent phase. Solvent clustering and preferential binding effects could plausibly occur in brushes in equilibrium with a vapor, although both are tied to specific compositions of the sorbed solvent; for preferential binding, the preferred solvent must be the minority component, whereas cluster formation restricts the composition of the sorbed solvent depending on the cluster stoichiometry. Hence, tuning of the vapor composition in addition to the polymer and solvent chemistry may be required to produce cononsolvency. Recent experiments in the Müller–Buschbaum group do indeed show cononsolvency for thin nongrafted films of a PNIPAm-based block copolymer, a PNIPAm analogue and polysulfobetaine in a mixed water–methanol atmosphere.^86,103^ While the films investigated in these experiments still swell relative to their dry state, their thickness at intermediate compositions is less than in either pure vapor, indicating a cononsolvent effect.

#### 2.4.1 Outlook on Mixed Vapors

As discussed in this section, binary liquid mixtures can cause counterintuitive swelling responses already. While these responses are reasonably well-understood by now, there is little evidence of their occurrence in gaseous environments. While our qualitative arguments suggest that cononsolvation by vapors is likely to exist, and cosolvation is not completely precluded, a more rigorous theoretical approach to extending liquid-based models could inform experimental research on these phenomena. Additionally, further experimental evidence of cosolvation and cononsolvation by vapor would be of technological interest due to the possible use of vapors as a switching mechanism. Experimental studies on this subject may be complicated by differences in volatility between solvents, however. On the technical side, fine control of the vapor composition across a wide range of densities may be required. Additionally, the potential difference in volume fractions between the components of a mixed vapor may lead to different kinetics of sorption for each component, requiring careful monitoring to ensure an equilibrium state is reached. Finally, in many applications vapors will be composed of many more different components. It is probable that many of these components are poor solvents—for instance, dry air generally causes polymer brushes to collapse—and hence, sorption will be dominated by components for which the brush is selective or which are present at near-saturated concentrations. However, complex partitioning of minority components may occur as a result of collaborative absorption and the respective miscibilities of the many species present in such a system.

### 2.5 Mixed Brushes

In this section, we discuss mixed polymer brushes: brushes consisting of two or more distinct types of polymer chains. Such brushes can form a range of different structures depending on solvent conditions, compatibility of the polymers, composition of the brush, and other parameters.^104^ This results in switchability of the surface composition and structure, which provides additional avenues for control of surface properties. Here, we provide an outline of these structures and their responsiveness to solvent vapors.

**Figure 8:**
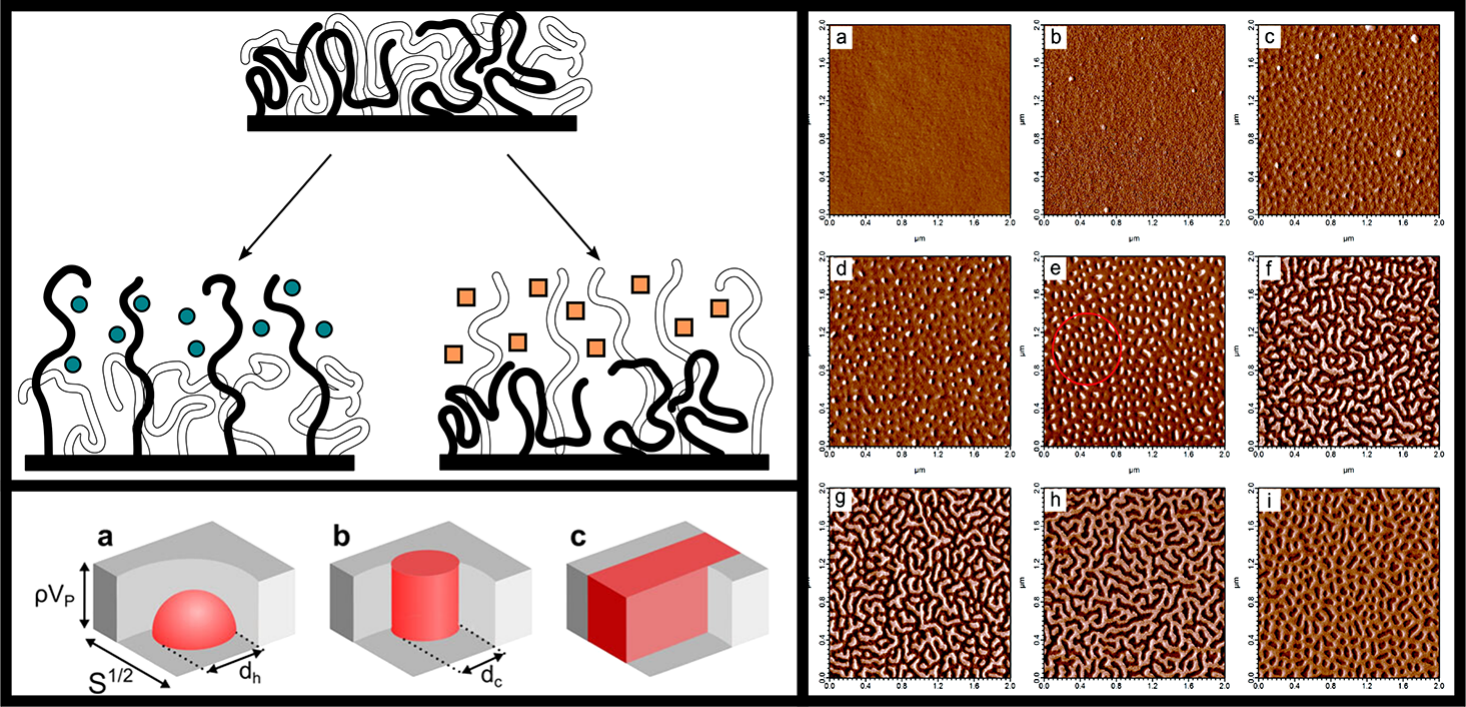
Phase segregation in mixed polymer brushes. Top left: Mixed polymer brushes may vertically separate when the surrounding medium is selective for either polymer species, altering the surface properties. Bottom left: Mixed brush systems may laterally segregate into (a) hemispherical, (b) cylindical, and (c) elongated stripe domains. Reprinted with permission from ref (111). Copyright 2021 AIP Publishing. Right: atomic force microscopy (AFM) images of lateral phase separation in PS/PMMA brushes annealed under tetrahydrofuran vapor. PS content increases from 0 to 68% by volume from part a to part i. The red circle in part e denotes a region of cylindrical domains that may be hexagonally ordered. Reproduced with permission from ref (112). Copyright 2012 American Chemical Society.

Two polymer species grafted to a substrate at sufficient densities will form a brush just like a single species would. When both polymers are similar, i.e., they are miscible and have similar affinities for the substrate and the free interface, this simply results in a brush structure in which the two chain types are randomly intermixed. However, dissimilarities between chains lead to phase separation. When the polymers are compatible but differ in their affinity for the substrate or the environment outside the brush, the two polymer types will assume different conformations, and a layer enriched in one of the two polymers may form at the interface, as shown in Figure 8.^105−107^ Zalakain et al. showed that rearrangements in mixed polystyrene/poly(methyl methacrylate) (abbreviated PS and PMMA respectively) brushes can be triggered by exposure to selective solvents, with acetic acid producing a PMMA-enriched top layer and cyclohexane enhancing the PS content of the brush surface, although in both cases contact angle measurements suggest that the surface layer still contained chains of both species. A degree of selectivity was also observed for vapors of these solvents.^105^ This selectivity is of interest for switchable surface functionalization and sensing applications. Klushin et al. point out that the presence of the other brush species turns the collapse-swelling transition of the polymer that swells in a given solvent from a continuous transition into a sharp one, enhancing the responsivity of the system, and present a self-consistent field theory of this phenomenon.^108^ In order to optimize responsiveness for a single analyte, having a minority of chains of the responsive polymer, with a larger average chain length than the nonresponsive polymer appears to be most favorable.^108,109^ Experimental work by Motornov et al. provides an interesting example of mixed brush responsivity in relation to gaseous environments specifically; in this study, a mixed poly(dimethylsiloxane)/ethoxylated poly(ethylenimine) (abbreviated PDMS and EPEI respectively) brush was found to be hydrophilic when submerged in water, but hydrophobic in air even at high relative humidities.^110^ This was attributed to the formation of a hydrophobic PDMS layer on top of hydrophilic EPEI clusters, which prevented spreading of water drops on the surface even when EPEI chains penetrated through the PDMS shell underneath the drop. This contact line pinning phenomenon is specific to three-phase systems, and it illustrates once again that brushes in gaseous environments display interesting properties beyond those that can be extrapolated from liquid-solvated brushes.

In addition to vertical separations resulting from collapse-swelling transitions of polymers, two incompatible polymer species in a brush may also phase-separate laterally. However, separation over large length scales is made impossible by the fact that the chains are anchored to a surface, leading to the formation of microdomains similar to those seen in block copolymer films. Self-consistent field studies and experiments both show that in binary brushes, these microdomains can take the form of a ”ripple” phase, with extended highly directional domains, or hexagonally packed cylindrical or hemispherical domains (see Figure 8).^104,113^ Simocko et al. identify a conceptually similar, but more complex, phase diagram for ternary mixed brushes.^114^ Due to the ability to form regular features on nanometer scales, these structures are considered interesting for lithographic applications.^115^ Switching from a disordered or vertically segregated state to a lateral microdomain state can generally be achieved by treatment with a solvent vapor that is nonselectively good for both polymers.^107,112^ Santer et al. observed persistence of the domain structure across multiple cycles of selective and nonselective solvent vapor treatment in a PS/PMMA system, describing this phenomenon as partial domain memory.^107^ A followup study showed a far weaker memory effect for Y-shaped brushes, which were produced using a surface-anchored bifunctional initiator. This memory effect was attributed to variations in the local composition of grafted chains, which are largely eliminated by the use of these Y-polymers.^116^ Eliminating this domain memory effect might enable the use of patterned brush surfaces to transport selectively adsorbed particles around under repeated patterning and depatterning by solvent cycling. Bao et al. investigated mixed brushes grown using a Y-shaped initiator on silica particles, tuning the grafting density of the brushes via the initiator/particle ratio. Microphase separation was found to become stronger with increasing grafting density, whereas the typical width of the ripple microphase decreased.^117^ Finally, recent simulation studies in our group have shown that microphase-separated brushes display enhanced vapor absorption capacity and overall solvent affinity, as the polymer–polymer interfaces form a high-energy interface that readily adsorbs solvent vapors.^111,118^

#### 2.5.1 Outlook on Mixed Brushes

While mixed polymer brushes display a range of interesting properties, they share some of these with free or grafted coatings of copolymers, which may be easier to produce. Nonetheless, their mechanical stability and phenomena such as domain memory effects are of unique interest. Further research into the change of mixed brush conformations as a function of solvent conditions may be of interest, both to optimize brush architecture and chemistry for potential applications and to better understand the thermodynamic and kinetic effects of, e.g., vapor annealing. However, producing mixed polymer brushes of high grafting densities for a wide variety of polymers remains nontrivial, especially for immiscible polymers. Identifying flexible and robust synthetic strategies for producing mixed brushes is another substantial open issue.

## 3 Applications

### 3.1 Sensing

**Figure 9:**
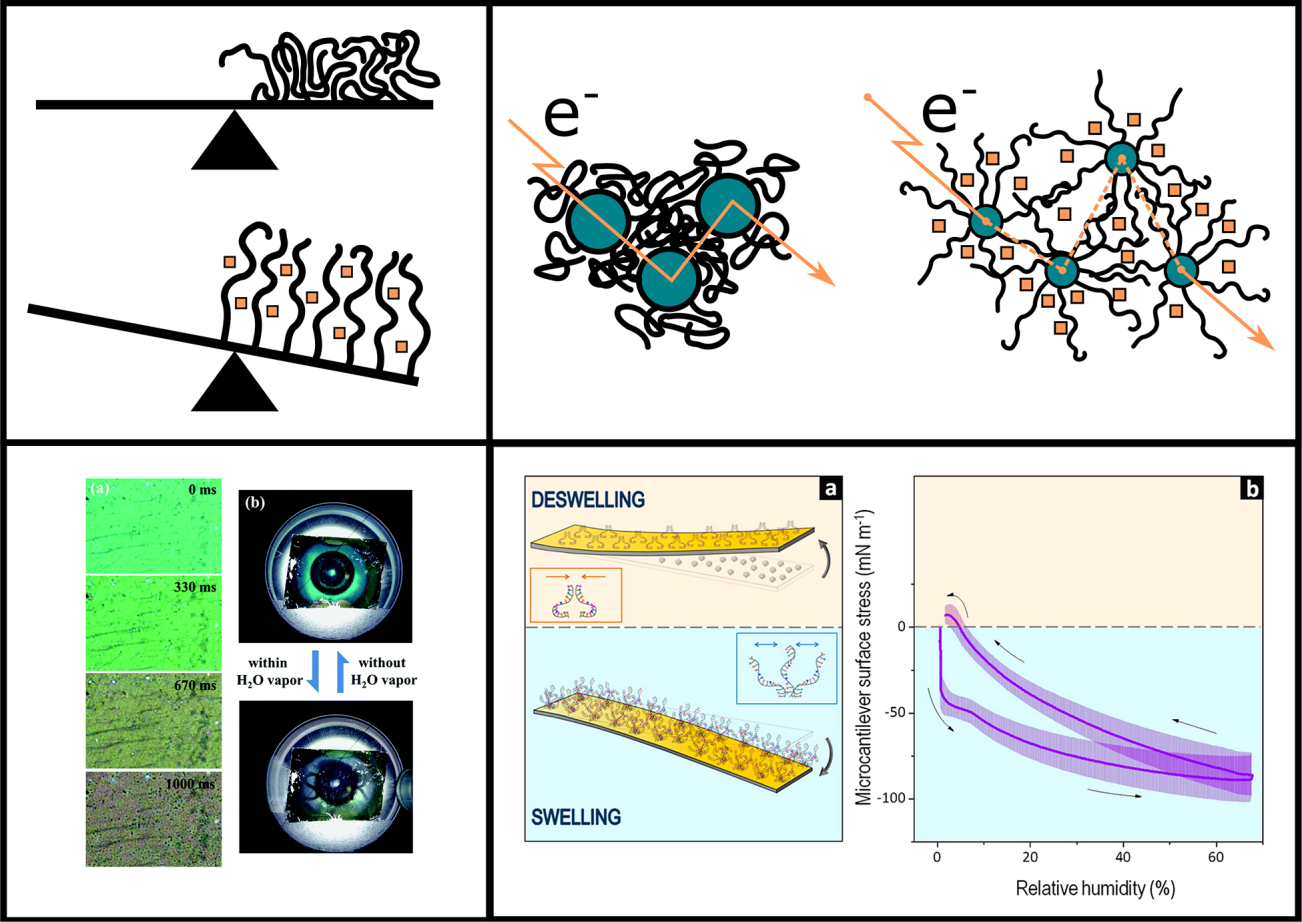
Variety of sensing methodologies incorporating polymer brushes. Top left: solvent vapor uptake by polymer brushes can be detected gravimetrically. Typically, these microscopic mass changes would be measured via the shift in resonant frequency of an oscillating system, such as in a QCM setup. Top right: The swelling of grafted polymers on conductive particles can break up conductive paths, altering the electric resistance of nanoparticle–brush composites. Bottom left: Swelling of polymer brushes can alter active length scales in optically active materials, leading to a change of color. Shown here: solvent uptake in polymer brushes on the bottom of silver nanovolcano arrays lead to a shift in color from light to dark green. Reproduced with permission from ref (119). Copyright 2017 Royal Society of Chemistry. Bottom right: swelling of grafted polymer chains or DNA strands increases lateral stresses within the brush, which can lead to bending of thin or soft substrates. Reproduced with permission from ref (70). Copyright 2014 American Chemical Society.

The stimulus-responsive nature of polymer brushes makes them of great interest for a wide range of sensing applications. In this section, we discuss how solvent-absorbing brushes can be used to enable and improve sensing technologies for chemical detection in the gas phase specifically. We include both designs which use brushes to enhance the capabilities of a separate sensing platform and ones in which the brush response itself (directly or indirectly) measures the analyte concentration. Vapor sorption in polymer brushes can generate or amplify a sensor response in a variety of ways, some of which are illustrated in Figure 9.

#### 3.1.1 Gravimetric Sensing

In gravimetric sensing techniques, the concentration of an analyte is typically measured by the shift in resonant frequency of a piezoelectric component as its mass changes with the adsorption of analyte. Grafting polymer brushes onto the resonating component in such a setup can increase the affinity between the solvent and the sensor surface, the selectivity toward the analyte, and the total sorption capacity. For example, McCaig et al. modified piezoelectric silicon nitride cantilevers with both grafted and drop-cast PMMA, and recorded the shift in resonant frequency of these cantilevers on exposure to various organic vapors. The response of the brush-coated cantilevers relative to the bare and drop-cast ones was enhanced substantially in polar vapors, which are generally compatible with PMMA, but was not altered significantly for apolar vapors.^22^ This approach also applies to quartz crystal microbalance (QCM) setups, in which the resonant frequency of a piezoelectric quartz crystal is monitored.^61,120,121^ Brush-enhanced QCM has been applied for a variety of polymer–solvent systems, with varying response kinetics and reversibility. High degrees of tunable selectivity are attainable by functionalizing the brushes with specific side groups. Kimura et al. demonstrated this by using metallophtalocyanines with different steric protecting groups, which resulted in different selecitivities toward various volatile organic compounds.^121^

#### 3.1.2 Electronic Sensing

Brush-based compounds can also be used in electronic sensors. Typically, this is done by coating conductive particles with a polymer brush layer, so that electronic contact between particles becomes dependent on the swelling state of the brush. Dispersions or layers of such brush-coated particles will show increasing electric resistivity as the brush swells, creating another method of translating a brush response into a signal. For instance, Li et al. demonstrated that grafting polymer chains onto graphitic carbon nanofibers (GCNFs) enhanced the response of a GCNF/platinum interdigitated array electrode to various vapors by at least an order of magnitude relative to bare GCNFs on the same electrode. Moreover, this enhancement was found to increase with the polymer–solvent affinity, creating a degree of selectivity.^21^ Wang et al. showed that the resistivity of dip-coated thin films of carbon black (CB) particles functionalized with polystyrene and poly(4-vinylpyridine) brushes increases strongly in the presence of good solvent vapors, as the swelling of the polymer disrupts the conductive network.^122^ This response was found to be reversible for methanol vapor, with larger alcohols producing a residual resistivity. Previously, Chen and Tsubokawa demonstrated that a similar concept, in which CB particles were dispersed into a nongrafted polymer matrix, could also be improved for a range of good solvent vapors by surface functionalization of the CB with brushes. In this case, the brush coating increases the dispersibility of the particles by lowering the surface energy of the carbon black, and improves the response time and reusability of the sensor material by preventing vapor molecules from binding directly to the carbon black surface.^123,124^

#### 3.1.3 Optical Sensing

Last, we highlight the use of polymer brushes in optical sensors, in which the solvent response of the brush alters the interaction between some part of the sensor and incident light. This is a rather diverse category, as most sensor designs under this umbrella are based on intrinsically optically active structures, in which a brush is used to vary the optically relevant dimension. Wei et al. grew multiresponsive polymer brushes on a gold substrate, and further coated this brush layer with a gold top layer to produce an optical cavity akin to an etalon. The size of this cavity depends on the thickness of the brush layer, and so the reflected wavelengths change as the brush responds to shifts in temperature, pH and relative humidity.^125^ Wang et al. created optical responsiveness using a silver nanovolcano array, an optically active structure of open, truncated cones,^126^ with a PNIPAm brush coating at the bottom of the nanovolcano cones.^119^ Such nanovolcano arrays are monochromatic transmitters, in which the vapor swelling of the PNIPAm brush produced a shift in color by altering the effective depth of the cones. Another optical sensor design measures the deflection of a laser by a brush-coated substrate, thereby detecting the bending of the substrate. For sufficiently thin or soft surfaces, a brush can relax the lateral excluded volume stresses associated with swelling by bending its substrate rather than by chain stretching.^127^ This property was utilized by Domínguez et al., by creating films of grafted single-stranded DNA with specific sequences on gold microcantilevers. When exposed to complementary DNA fragments in solution, these grafted DNA strands hybridize to form double-stranded DNA, which is significantly more rigid than ssDNA.^70^ This results in a void-filling swelling mechanism, with less variation of lateral stresses and hence a different bending response.^32^ This has been proposed as a rapid and tunable detection technique for identifying pathogens or specific genetic sequences, such as the ones responsible for antibiotic resistance.^70,128,129^

#### 3.1.4 Outlook on Sensing

The responsive and selective nature of brushes gives rise to a wide range of potential sensor designs. However, some challenges present themselves. In addition to the general issues of brush stability and scalable synthesis, the actual range of applications for brush-based sensors is limited by the analytes for which brushes can be made sufficiently selective. Chemical research to tailor polymers to specific target compounds could expand the applicability of brush-based sensing concepts. Additionally, fouling or interactions between common gas components could influence the sensing functionality, and they may therefore be worth investigating.

Finally, some sensor designs for liquid environments may also work under gases. For instance, in one study, opal-like arrays of silicon nanoparticles coated with brushes containing hydrophobic and negatively charged blocks were shown to change in color in the presence of lysozyme proteins, due to the change in Bragg reflection wavelength as the brush swelling alters the periodicity of the structure.^130^ While this was demonstrated in a liquid environment, a solvent vapor in air is from a thermodynamic perspective comparable to a minority component with strong polymer affinity in a poor background solvent, suggesting that concepts like this may extend to the gas phase as well.

### 3.2 Separations

Polymer brushes’ potential for selective absorption has the potential to enhance various separation technologies. Additionally, hydrophilic polymer brushes have found use in forming conducting channels with applications for proton separation or in enhancing the formation of such channels in existing proton separation materials. In this section, we provide an overview of these applications and the role polymer brushes play in them.

#### 3.2.1 Gas Separations

**Figure 10:**
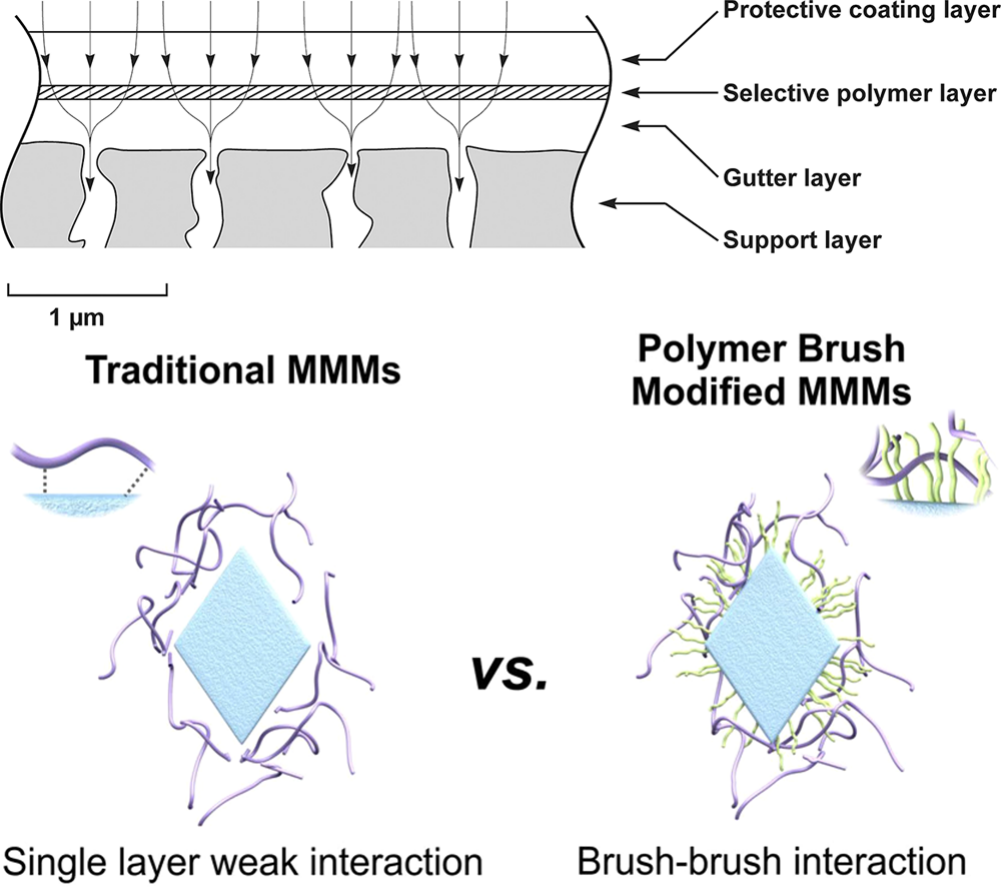
Incorporation of (grafted) polymers in membrane architectures. Top: Typical membrane architecture, consisting of a protective coating, a thin selective layer, and a porous, typically inorganic support. A ”gutter layer” may interspersed to compatibilize the support and the selective layer. While we focus on the use of grafted polymer materials in the selective polymer layer, membrane properties can also be modified by anchoring polymer chains inside the porous support. Reproduced with permission from ref (131). Copyright 2017 American Chemical Society. Bottom: contrast between grafted and nongrafted mixed matrix membrane (MMM) structures. Usually, MMMs consist of separate inorganic particles incorporated in a polymer matrix. However, functionalizing the particle surface with a polymer brush can improve the compatibility of the surface and the polymer matrix, and prevent particle aggregation. Reproduced with permission from ref (132). Copyright 2018 American Chemical Society.

The most broadly relevant gas separations are between combinations of N_2_, O_2_, H_2_, CO_2_, CH_4_, and He. Some notable combinations of these comprise air separation, natural gas sweetening, flue gas treatments, and hydrogen separation.^133−137^ Membranes used in these separations classically consist of a thin, highly selective layer over a porous support material, which provides mechanical stability (see Figure 10). For sufficiently large pore sizes, surface functionalizations can also be applied to the inside of the pores in the support material.^138^ Research on this class of polymer brush-based membranes has been reviewed by Keating et al.^10^ Bruening et al. provide an overview of the synthesis and use of polyelectrolyte multilayers and polymer brushes for membrane applications, and they conclude that their selectivity and compatibility with a range of supports makes polymer brushes an attractive class of materials for gas separations.^138^ For example, Balachandra et al. investigated the performance of poly(ethylene glycol dimethacrylate) (PEGDMA) and poly(hydroxyethyl methacrylate) (PHEMA) brushes anchored from polyelectrolyte multilayers on porous alumina.^135^ They report high CO_2_ permeability and selectivities toward CO_2_ for the PEGDMA-functionalized membranes, which is attributed to cross-linking of the PEGDMA brush (which favors the diffusion of small molecules) and high solubility of CO_2_ in the brush through polar interactions with carbonyl groups in PEGDMA. PHEMA, on the other hand, did not display any significant selectivity. Upon functionalization with a perfluorinated side chain, however, the PHEMA-based membrane acquired CO_2_ permeability comparable to the PEGDMA-functionalized membrane, albeit with lower selectivities toward CO_2_. This suggests functionalized PHEMA layers with an appropriately selected side group may be of use for specialty separations. Grajales et al. show that membranes functionalized with brushes of poly(ethylene glycol methyl ether methacrylate) (PEGMEMA) polymers display enhanced CO_2_/H_2_ selectivity when PEGMEMA monomers with a variety of poly(ethylene glycol) (PEG) side chain lengths are incorporated. This dispersity of side chain lengths inhibits the crystallization of the brush, which would favor diffusion of the smaller H_2_.^139^ Aliyev et al. found that grafting PDMAEMA brushes to graphene oxide (GO) on a porous polyacrylonitrile support covered up pinhole defects in the GO layer and provided strong selectivity toward water vapor, enhancing membrane performance relative to a bare GO membrane.^140^

**Figure 11:**
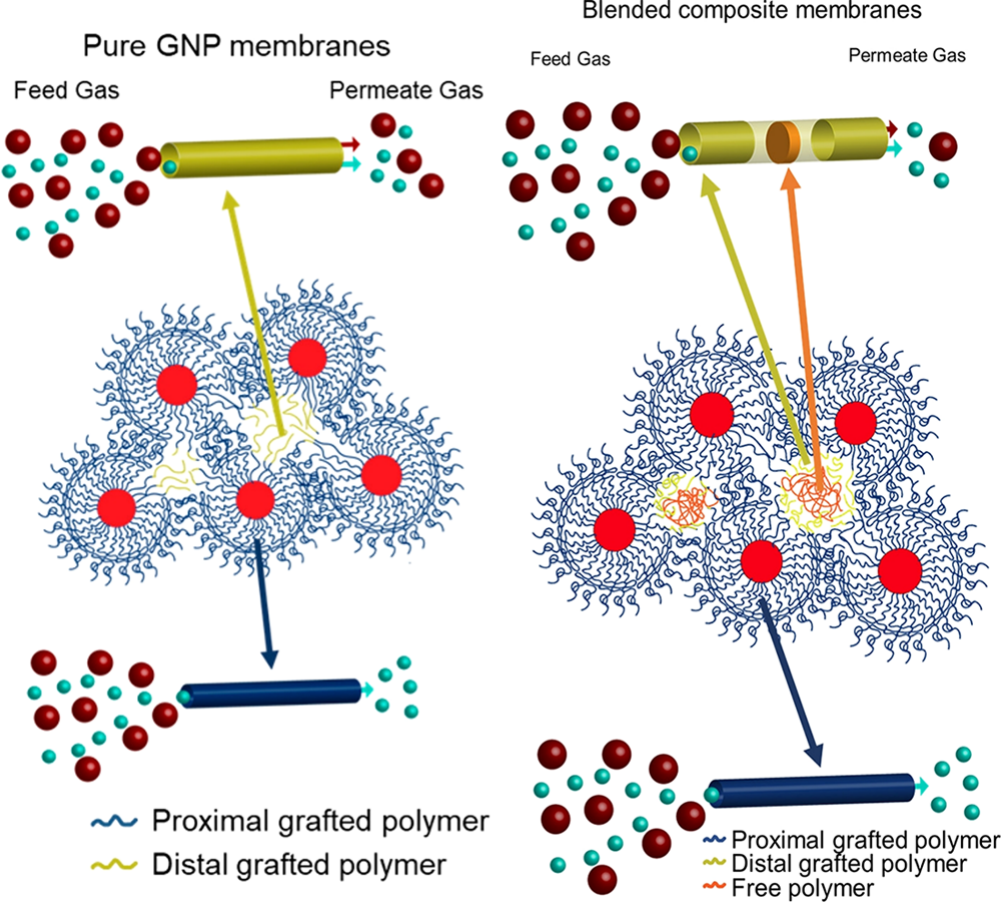
Inefficient packing of polymer chains in interstitial volumes reduces size-dependent selectivity in grafted nanoparticle membranes (left). Adding free polymer chains to this structure reintroduces a degree of size-based selectivity, dependent on the distribution and molecular weight of the free polymer. Adapted with permission from ref (141). Copyright 2020 American Chemical Society. .

Mixed matrix membranes, in which the polymeric layer is loaded with filler materials such as zeolites or metal–organic frameworks (MOFs) may improve upon the properties of the pure polymeric membrane functionalizations described above, e.g. by acting as molecular sieves or selective adsorbents.^131,142^ However, such materials are faced with stability issues such as aggregation of filler particles. When this architecture is modified by replacing the free polymer matrix with a grafted polymer coating on the particle surface, the dispersibility of the particles is improved.^141,143^ Moreover, Bilchak et al. showed that membranes composed of grafted silica nanoparticles displayed increased free volume relative to the neat polymer matrix, since the packing of the spherical particle leaves interstitial volumes, resulting in increased permeability at the expense of selectivity, as illustrated in Figure 11.^143^ Followup research showed that this free volume can be tuned through the addition of long free polymer chains which preferentially occupy the interstitial volume, reintroducing size-based selectivity to the membrane.^141^ Experiments by Jeong et al. using poly(butyl methacrylate)-grafted particles show that high grafting densities are required for increased gas permeability.^144^ This is attributed to the formation of polymer bridges between particles at low grafting densities, either during polymerization or by penetration of polymer chains from one particle to the surface of another, which can result in inhomogeneous dispersion of the particles. Xin et al. found that brush functionalization with polystyrene-derived polymers improved the compatibility of various inclusions with a sulfonated poly(ether ether ketone) (SPEEK) matrix.^145−147^ Additionally, they observed that pyridine-functionalized graft polymers enhance selectivity toward CO_2_,^145,146^ presumably due to the chemical similarities to amines, which are highly effective at binding CO_2_.^148^ Wang et al. compared a mixed matrix membrane design of metal–organic framework particles in a polyimide matrix with a membrane architecture composed purely of brush-grafted MOF particles, and similarly found that both membrane performance and mechanical stability are enhanced by improvement of the particle–polymer interfacial interaction. At high MOF loading, however, the increased viscosity of the grafted polymer relative to the nongrafted matrix hinders the solution casting procedure employed in this work, resulting in deterioration of membrane properties.^132^

#### 3.2.2 Proton Conduction

Polymeric membranes also find use in ion exchange membranes for energy applications, such as proton exchange membrane (PEM) fuel cells. In such fuel cells, hydrogen is catalytically oxidized at the anode, and the resulting protons diffuse through a membrane to react with oxygen and electrons at the cathode side. The archetypal polymer for proton exchange membranes is Nafion, a perfluorinated polymer with ether-linked sulfonated oligomer side chains, which phase-separates under humid conditions to form hydrophilic channels with a high density of sulfonate groups, which are suitable for selective proton transport.^149,150^ However, Nafion and similar perfluorinated polyelectrolytes are costly, require high humidity to function, and are only moderately stable mechanically. Farrukh et al. incorporated silica nanoparticles functionalized with grafted poly(monomethoxy oligo(ethylene glycol) methacrylate) (PMeOEGMA), a hygroscopic polymer. Small amounts (1 wt %) of these nanoparticle inclusions were found to enhance proton conductivity by up to an order of magnitude, with the largest improvements observed at low temperatures and humidities.^151^ Niepceron et al. fabricated membranes of an inert fluoropolymer matrix with poly(styrenesulfonate)-grafted nanoparticle inclusions, and found that these composites displayed high proton conductivities in addition to self-humidifying properties.^152^ Most research on grafted polymers for ion exchange membranes focuses on nonfluorinated polymer sulfonates, however. Yameen et al. investigated membranes composed of polyacid brushes on macroporous silica, and found that these materials displayed proton conductivity approaching that of Nafion membranes and enhanced mechanical stability due to the presence of a solid scaffold.^149,153^ Incorporating hygroscopic PMeOEGMA blocks in the polymer brush in these same membranes reduced the temperature and relative humidity dependence of the proton conductivity, leading to high conductivity under a wide range of conditions.^154^ Another research team performed several studies in which partially sulfonated polystyrene was grafted onto filler materials and dispersed these into a polymer matrix. This generally results in enhanced proton conductivity relative to the neat polymer.^155,156^ The improved conductivity is generally attributed to improved dispersibility of the filler and formation of conductive networks throughout the polymer matrix.^156^ Zheng et al. investigated the effect of polymerization parameters on the conductivity of poly(2-acrylamido-2-methylpropanesulfonic acid) (PAMPS) brushes grafted to titanate nanotubes, and found nonmonotonic dependencies on both grafting density and chain length.^157^ Dong et al. studied a similar system, consisting of PAMPS brushes in aligned titanate nanotubes, and report that the PAMPS brush enhances proton conduction relative to the bare nanotube array. This enhancement is observed when nanotubes are partially or completely filled by the brush, suggesting that sulfonate groups near the nanotube wall have the largest impact on conductivity.^158^ However, we point out that incomplete pore filling may have detrimental effects on selectivity.

#### 3.2.3 Outlook on Separations

As described above, many brush-based solutions for separations have been proposed. Yet, we see room for improvement. A systematic theoretical study of the effects of brush characteristics (grafting density and polymer length) on the gas transport properties has not been performed so far and this might be key in optimizing the separation performance. Additionally, in some of the works discussed in this section, polymer brushes are used primarily to compatibilize inorganic components with the polymer matrix in a mixed matrix membrane architecture. In these cases, significant improvements can be made on a long-term stability and the prevention of particle aggregation. Extrapolating from existing works, the use of cross-linking functionalities to covalently bond particles to the matrix could be of interest here. While this would not necessarily reduce the particle–polymer interfacial energy relative to nonbonded brush-bearing particles in the matrix, it would create additional physical barriers to particle aggregation and enforce matrix-particle contacts. In other cases, such as the majority of proton separation applications we describe, the grafted polymers qualitatively alter transport through the membrane. The directionality imposed by grafted polymer, the high density of specific binding sites, and transport along brush–air and brush–inorganic interfaces are all potential contributors to permeability and selectivity. Further research into the physical structure of membrane architectures containing grafted polymers could indicate which of these contributions are most significant and how membrane architecture can be further optimized for this.

### 3.3 Adhesion and Friction Control

The unique structure and solvent-binding abilities of polymer brushes lead to interesting mechanical properties in addition to the previously discussed chemistry-oriented applications. In this section, we describe the friction and adhesion properties of polymer brushes, and we outline how these responses are modified by the presence of solvent vapors. Additionally, we highlight experimental works that make use of these properties and theoretical approaches that investigate the specific effects of vapor solvation.

#### 3.3.1 (Non-)Selective Adhesion

Adhesion is a surface property with clear practical applications: highly adhesive surface coatings could be used as glues, whereas low adhesion makes surfaces fouling-resistant and easy to clean. Adhesion is typically defined by the reduction in surface energy upon putting two surfaces together. Hence, surfaces that interact unfavorably with the background medium tend to be nonselectively adhesive. A classic polymeric example is the hydrophobic polymer PDMS, a commonly used material in microfluidics for biomedical applications, which is hindered by its tendency to nonspecifically adsorb organic compounds in an aqueous environment. However, surfaces can also be tuned to interact strongly with specific materials through, e.g., hydrogen bonding or supramolecular chemistry, leading to selective adhesion. Additionally, the mechanical properties and nonideality of a surface may influence the adhesive properties: soft materials may conform to the contacting surface, whereas surface roughness may increase or decrease the effective contact area for soft and hard countersurfaces, respectively. Since the mechanical properties and surface energy of polymer brushes vary with the chain conformation, which can be tuned and switched by a wide variety of parameters, polymer brushes are of great interest for modifying surface adhesion.

In many settings, preventing the adhesion of certain components to a surface is important. This includes precipitation of salts and other solids in industrial contexts, protein and cell adhesion in biomedical applications, and growth of both micro- and macroscopic organisms in marine environments. This is generally achieved by antifouling surface functionalizations. In a recent review on antifouling polymer surfaces, Maan et al. distinguish three forms of antifouling functionality: fouling resistance, in which adhesion of certain components is prevented, fouling release, in which foulants can weakly adhere to the surface but are easily removed by some external stimulus or force, and fouling-degrading, in which the material breaks down adsorbed foulants.^159^ In this framework, hydrophilic polymer brushes are naturally fouling-resistant due to their high polymer density, their internal osmotic pressure,^160^ and the formation of a tightly bound water layer around the polymers.^161,162^ While linear PEG brushes are a simple and commonly applied example,^163^ research into other polymers and brush architectures for antifouling is ongoing. Examples include the use of sugar-functionalized brushes to selectively promote and reduce bacterial adhesion^164^ or zwitterionic brushes for general protein repulsion.^165^ Variations in architecture can further enhance the coating properties. Wang et al. found that a bottlebrush coating of poly(*N*-vinylpyrrolidone) (PVP) attached to a PHEMA backbone was more effective at preventing protein adhesion to a gold surface than a linear PVP brush of the same thickness.^166^ Morgese et al. investigated loop-type brushes, consisting of cyclic polymers anchored to a surface by a single point.^167^ These loop brushes can accommodate higher areal densities of polymer than linear brushes, which could result in enhanced antifouling capacities. PDMS brushes have been identified as particularly effective antifouling coatings.^168−171^ Even oils that completely wet almost all substrates easily roll off substrates coated with PDMS brushes. This has been attributed to the low surface energy of PDMS and the intrinsic liquid state of these brushes.

**Figure 12:**
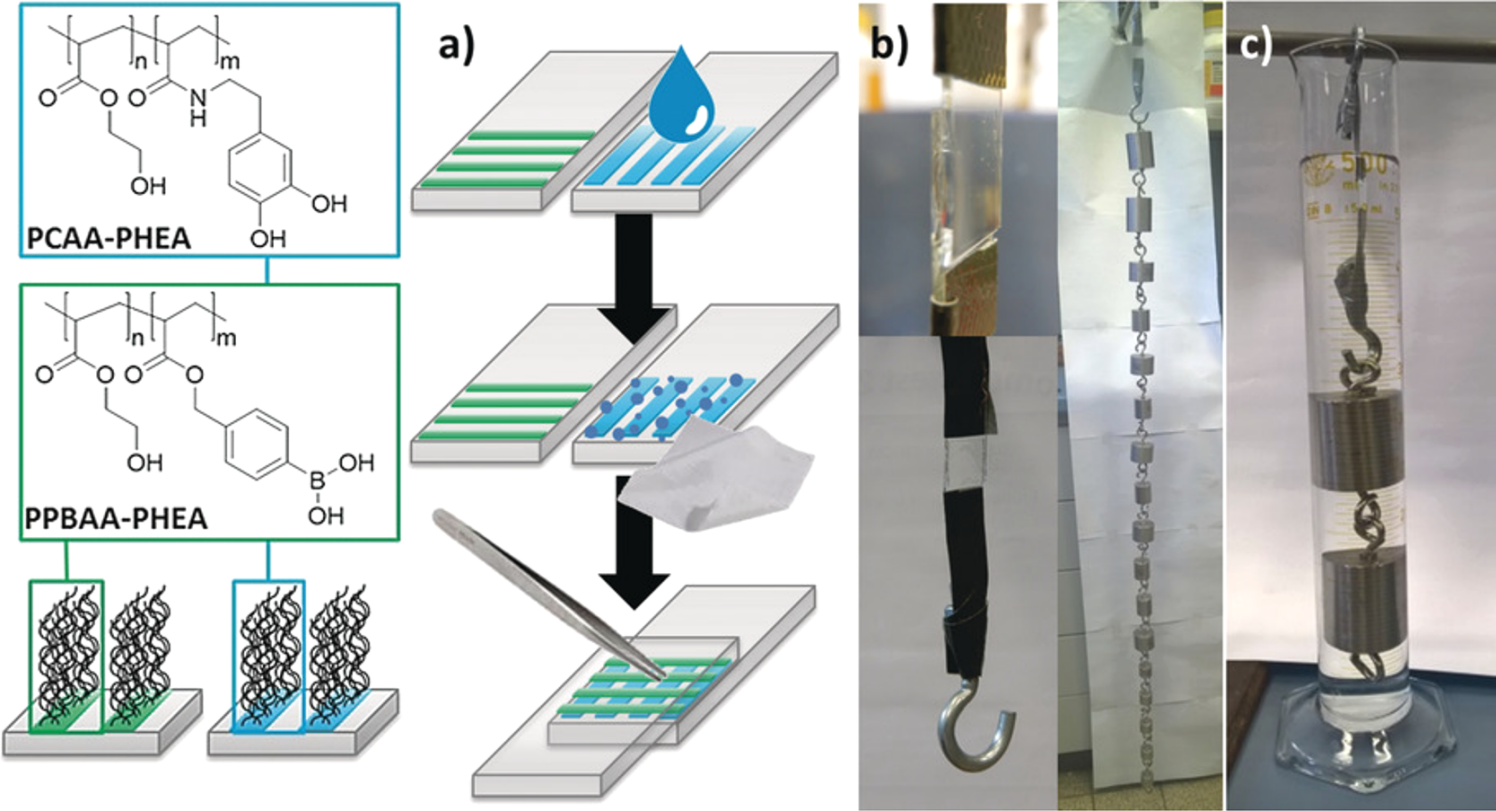
Polymer brushes can serve as an effective platform for supramolecular adhesives. Shown here are copolymers of phenylboronic acid acrylate (PPBAA) and catechol acrylamide (PCAA) with hydroxyethyl acrylate (PHEA). (a) After preparation of the brush-coated surfaces, a drop of deionized water is placed on one of the surfaces, left to rest for 10 min and dried with tissue paper before placing the surfaces together for 30 min. This provides a favorable environment for the formation of dynamic covalent bonds between the boronic acid and catechol functionalities.^173^ (b) Close-up of adhering surfaces and weight test using a chain of weights hanging on the glued surfaces. (c) Similar weight test performed in water, indicating the water-resistant nature of the adhesive. Reproduced with permission from ref (173). Copyright 2018 Wiley-VCH Verlag.

Besides reducing unwanted adhesion, polymer brushes can also be used as general or targeted adhesives by presenting a high density of functional groups. Chaudhary et al. studied the adhesion of PDMS surfaces functionalized with poly(2-ethylhexyl acrylate) (P2EHA) brushes by recording force–distance curves with a sapphire probe.^172^ While this functionalization did enhance the adhesion between the PDMS and the probe, a maximum in the adhesion as a function of chain length was observed. This maximum was attributed to longer P2EHA chains entering into and stiffening the PDMS network. Nonetheless, the results for lower chain lengths illustrate the ability of hydrophobic polymers to function as dry adhesives. Supramolecular compounds based on multivalent host–guest interactions can function as a strong and selective adhesive, and are in many cases switchable. Lamping et al. have reported several examples of such supramolecular adhesives, in which the host and guest functionalities are attached to different brush coatings.^173,174^ In particular, a system based on the interaction between phenylboronic acid- and catechol-containing brushes was found to be strongly adhesive, water-resistant, and switchable by the addition of carbohydrates.^173^ As illustrated in Figure 12, once activated and placed together, these brushes performed well in weight tests.

#### 3.3.2 Switching Adhesion

Finally, adhesion modification is compatible with the switchable behavior of polymer brushes. Synytska et al. synthesized copolymer brushes with randomly distributed (hydrophobic) PDMS and (hydrophilic) PEG side chains.^175^ These brushes were found to display a degree of phase separation, resulting in an enrichment in PDMS at the brush–air interface under dry conditions and an enrichment of PEG when submerged in water. Adhesion forces with both hydrophilic and hydrophobic probes were found to be nonlinear with the brush composition, with higher PEG content leading to higher adhesion to hydrophilic surfaces. However, PEG-rich systems fully submerged in water displayed low adhesion regardless of the probe, presumably due to a preference for PEG-water contacts.

#### 3.3.3 Dissipation and Friction Mechanisms

In an idealized scenario, adhesion is thermodynamically reversible: the reduction in surface energy when putting two surfaces together should be equal to the energy required to separate them. However, in reality, separating the surfaces typically requires some additional energy. This phenomenon is called adhesion hysteresis, and is generally related to rearrangements in the material upon contact or separation. An intuitive example in soft materials is deformation to maintain contact with the countersurface, which dissipates additional energy. This adhesion hysteresis can be intuitively related to friction: an object moving over a surface is continuously making contact with the surface ahead of it, while separating from the surface behind it. With this in mind, we look at the ways this phenomenon manifests in polymer brushes and how this can be used in order to modify surface friction.

**Figure 13:**
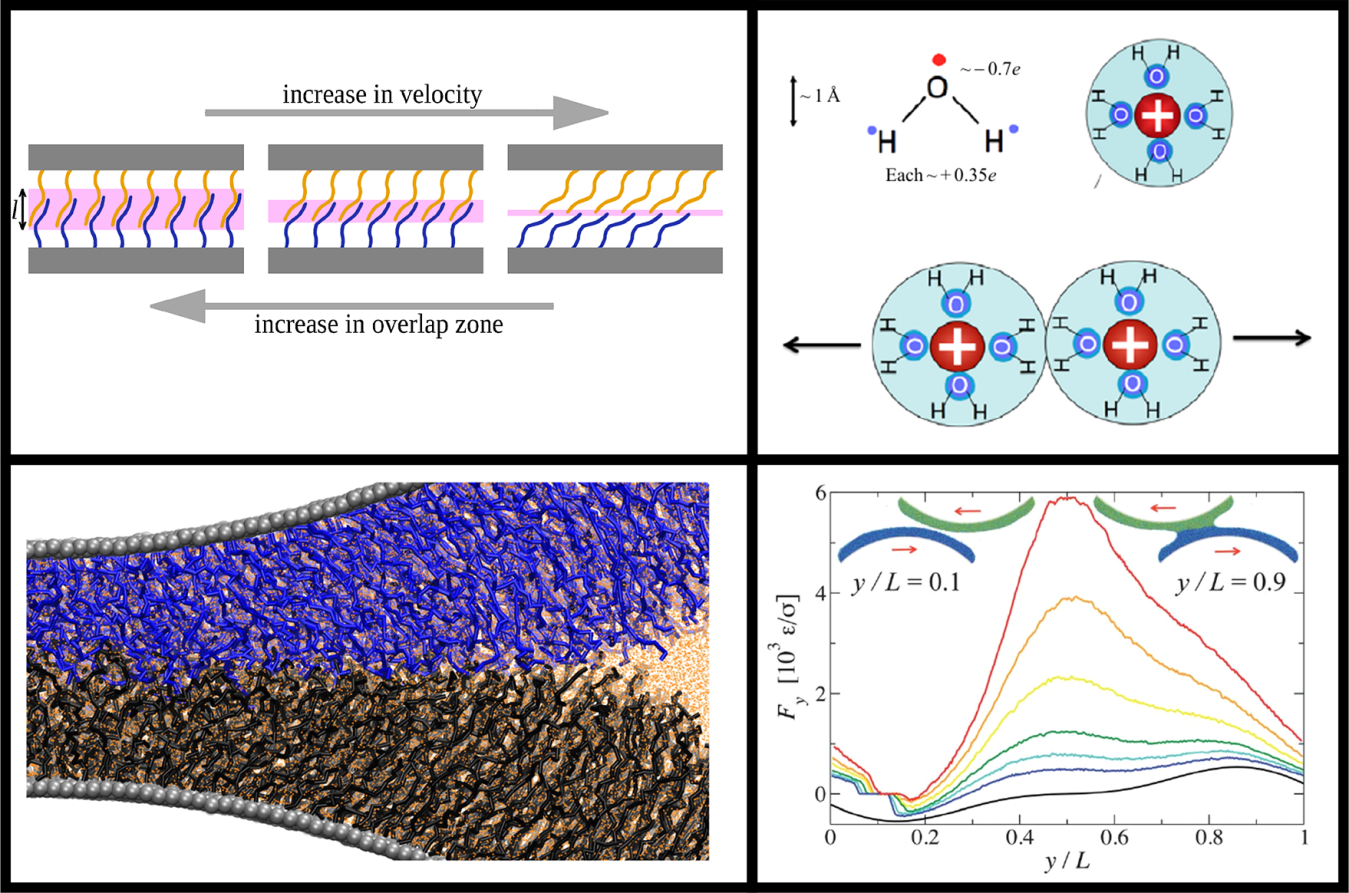
Selection of relevant mechanisms in friction and lubricity in polymer brush bilayers. Top left: Reduction of the interdigitation zone by chain tilting under shear. Reproduced from ref (176), originally released under a Creative Commons attribution (CC-BY) license. Top right: repulsion between hydration shells around solvated polymers. Reproduced from ref (3), originally released under a CC-BY license. Bottom left: chain tilting out of the point of contact between asperities. Reproduced with permission from ref (177). Copyright 2014 American Chemical Society. Bottom right: formation and movement of a meniscus between the brush-coated surfaces. Reproduced with permission from ref (178). Copyright 2013 Royal Society of Chemistry.

Although the two phenomena are somewhat different in origin, many of the properties that lend polymer brushes their low adhesion are also relevant to their lubricious properties. The internal osmotic pressure of the brush creates an opposing force to compression^179^ and inclusions,^160^ reducing the degree to which a countersurface can be pressed into the brush and, consequently, the contact area over which friction forces apply. Brush bilayers, contacts between two polymer brushes, can also serve as effective lubricants: while the osmotic pressure in two identical brushes is the same, the entropic cost of chain stretching limits brush interdigitation to a relatively narrow region near the center of the bilayer.^180^ Since the interdigitation of chains in the contact area is an important source of adhesion hysteresis in brush bilayers, this helps reduce friction. Alternatively, this can be considered as a reduction in the effective contact area between the brush-covered surfaces. Moreover, the chains in a brush bilayer may tilt under shear, further reducing the width of the region where the brushes contact each other (see Figure 13, top left).^181^

Various types of dissipation mechanisms can determine the friction in brush bilayers in air, as was studied using molecular dynamics simulations. In these simulations, two opposing brush-coated cylinder sections are moved against each other in longitudinal, transverse, and normal motion, simulating shear between smooth surfaces, friction between colliding asperities, and compression.^178^ Solvent molecules are included in the brushes, but no bulk solvent phase is present outside the brushes, meaning that these simulations are comparable to experiments in air at high relative humidity. The obtained friction scalings indicate that asperity collisions are qualitatively different from smooth-surface shearing, as transient interdigitation^182^ of the polymer brushes becomes relevant in addition to steady-state interdigitation. Moreover, a meniscus forms between the two surfaces, creating adhesion hysteresis and thus friction as the point of contact moves over the cylindrical geometry and the meniscus changes shape. This phenomenon is particular to the system in air, and it shows that friction between rough surfaces in humid air is qualitatively different from that in water. In a later study, the same authors studied similar systems containing two chemically distinct polymer brushes and two solvent types. It was found that for liquid-immersed systems, the curvature of the cylindrical surface allows chains to tilt away from the point of contact between the asperities, reducing interdigitation and thus friction. Similar behavior holds for systems ”in air” when the two brushes are made immiscible, either by preferential absorption of immiscible solvents or by direct repulsion between the polymers. However, in miscible undersaturated systems, capillary contributions force the chains toward the contact and lead to increased friction.^177^ Further simulations and experiments also support that dissipation is restricted when the opposing polymer brushes are immiscible, leading to lower friction.^30^

#### 3.3.4 Lubricity

In addition to the various chain conformation and orientation effects discussed in the previous paragraphs, the interaction between polymer brushes and solvents contributes to the lubricity of swollen brushes, as demonstrated by Jacob Klein and co-workers in various works. Due to the aforementioned osmotic pressure, an organic solvent absorbed in a polymer brush resists squeeze-out even under high loads, and it ensures the fluidity of the brush in the contact region.^183^ Polyelectrolyte bilayers in water, however, display even stronger and more robust lubricity.^184^ This has been attributed to a combination of increased osmotic pressure as a result of the presence of counterions, which would enhance the previously discussed effects, and enhanced repulsive interactions arising from the hydration shell around the charged polymer segments (see Figure 13). The hydration shell is tightly bound and stable, while remaining liquid even at short time scales due to the rapid exchange of water molecules between the hydration shell and the surrounding liquid.^3,185^

Kobayashi et al. demonstrated low friction between a glass probe and a surface coated with the polyzwitterion poly(methacryloyl oxyethyl phosphoryl choline) (PMPC) in both water and humid air. Friction was found to decrease with relative humidity, and at high relative humidity (80%), the vapor-solvated system in fact displays lower friction than the liquid-solvated system.^28^ This was attributed to the liquid-solvated chains being more extended, allowing them to form a larger contact area with the probe. Friction in liquid toluene, a poor solvent, was found to be comparatively high. This may be due to polymer chains adhering to the probe in order to reduce unfavorable PMPC–toluene contacts. When both the probe and the brush were functionalized with PMPC brushes, creating a bilayer scenario, similar trends were observed for the water and humid air cases. However, the system submerged in toluene displayed slightly lower friction than the system in liquid water. This was attributed to the reduced interdigitation between poorly solvated (hence collapsed) brushes. Followup studies including polycationic and polyanionic brushes in bilayer geometries found that the friction in these systems was lowest when submerged in water, although swelling by water vapors did reduce the friction relative to the dry state.^186,187^ It remains unclear why, out of the polymers discussed in these works, only PMPC displayed lower friction in humid air than in water, although the original authors point out the possibility of lubrication by an adsorbed water layer due to the superhydrophilic nature of PMPC.^186^

Strongly self-interacting hydrophobic polymers can also function as lubricants, depending on the intended countersurface. Bhairamadgi et al. present results on both friction and adhesion on polymer brush coatings, in which poly(ethyl methacrylate) and poly(2,2,2-trifluoroethyl methacrylate) are compared. In all cases, the fluorinated polymer displayed substantially lower adhesion and friction with a silica probe than its nonfluorinated counterpart, with the most pronounced differences for adhesion under humid conditions.^29^ This was attributed primarily to the apolar, hydrophobic nature of the fluoropolymer, which reduces ab- and adsorption of ambient water and hence prevents the formation of a water meniscus between the brush surface and the probe. Adhesion and friction were found to decrease with increasing molecular weight and grafting density of the brush, an observation consistent with the fact that the compressibility of the brush also tends to decrease with these parameters.

#### 3.3.5 Switching Friction

**Figure 14:**
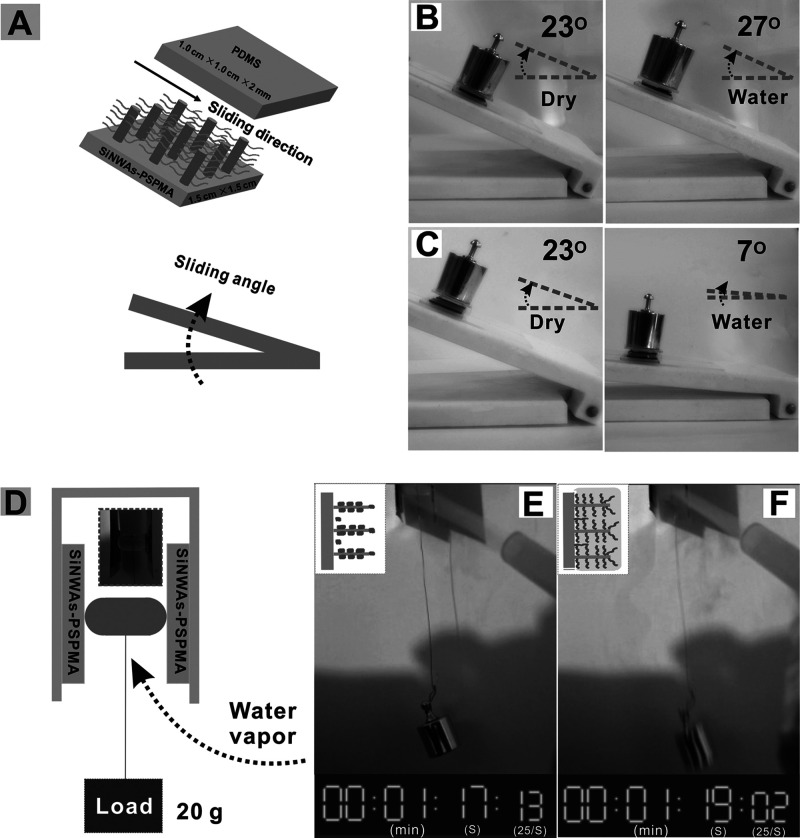
(a) In ref (188), the friction between a weighted PDMS surface and a PSPMA-functionalized silicon nanowire array (SiNWa) is tested by placing the system on an inclined plane and recording the angle at which the PDMS begins sliding. (b) Sliding angle measurements in dry air and in the presence of water vapor for the bare silicon nanowire array. (c) Sliding angle measurements for the PSPMA-functionalized SiNWa in dry air and in the presence of water vapor. The sliding angle in the presence of water vapor decreases significantly. (d) Alternative testing setup, in which a piece of PDMS with an attached load is clamped between two PSPMA-functionalized SiNWa surfaces. (e and f) When water vapor is introduced to the system, the clamped PDMS quickly slides downward under gravity, indicating a rapid friction response. Reproduced with permission from ref (188). Copyright 2014 Wiley-VCH Verlag.

A topic of technological interest for brushes in air is switchable friction. While we have already discussed the humidity dependence of friction behavior in various brushes, a range of other switching mechanisms is available. For example, friction can be switched by changing the degree of interdigitation by external stimuli.^189^ Moreover, Ma et al. demonstrated humidity-switchable friction of poly(sulfopropyl methacrylate) (PSPMA) brushes on a silicon nanowire array substrate (illustrated in Figure 14, and showed that the collapse of the PSPMA brush by increasing the salt concentration (“salting out”) can also be used to modify friction properties, with substantially increased friction for the salted-out brush.^188^ The same study also demonstrated pH-dependent switching in poly(methacrylic acid) brushes. Liu et al. realized switchable friction in polymer brushes under humid (90% RH) air by grafting a PSPMA brush from a matrix containing photothermally responsive Fe_3_O_4_ particles.^190^ Under near-infrared (NIR) irradiation, the thermogenic response of these particles resulted in the dehydration and collapse of the brush. This was paired with a change from low friction coefficients in the hydrated state, to high friction in the dehydrated state. Notably, rehydration upon switching off the NIR laser is rapid, on the order of seconds. Zeng et al. found that poly(allyloxy hydroxypropyl sulfonate) brushes in humid air can be reversibly collapsed using an external electric field.^191^ In their collapsed state, these brushes display reduced friction relative to their extended state. While the friction coefficients reported in this work are comparatively high, the demonstration of electro-switchable friction is of particular technological relevance.

#### 3.3.6 Outlook on Adhesion and Friction control

The general concepts underlying friction and adhesion in polymer brushes appear to be comparatively well-understood. As a result, many of the works reviewed here are focused on optimizing brush chemistry and architecture. However, the effects of relative humidity and the exact solvation state of the polymer will also impact friction and adhesion. As discussed in section 2.3, Goedel et al. showed that partially solvated brushes are parabolic (i.e., well-solvated) at the brush-air interface, but retain a constant density closer to the substrate.^82^ As a result, the outer surface of the brush may be considerably solvated even when the bulk is dry, resulting in a nonlinear effect of solvent uptake on the mechanical properties of the brush. Quantifying this will be of great interest for optimizing application conditions for brush-based adhesives and lubricants. Finally, due to the stresses inherently involved in mechanical applications, the stability of polymer brushes is a particular concern in friction control applications.

### 3.4 Wetting Control

As highly tunable surfaces with potentially switchable properties, polymer brushes can be used for tuning a wide variety of surface interactions. Controlling the wetting behavior of drops and liquid films on surfaces is an example that is specific to three-phase systems, most notably surfaces in air. While nongrafted polymer coatings can effectively modify surface interactions, surface anchoring provides stability under highly solvated conditions. Here, we discuss how polymer brushes can be applied to control surface wetting, and how their properties can lead to nonclassical wetting phenomena.

The classical description of partial wetting, i.e. drops on surfaces, is given by the Young equation γ_lv_ cos θ + γ_sl_ – γ_sv_ = 0, where the drop is considered as a spherical cap with a base angle of θ, γ_ij_ denotes the surface tension between phases i and j, and the subscripts s, l, and v indicate the solid surface, the liquid, and the surrounding vapor. When no value of θ satisfies this expression, either total wetting (θ = 0) or complete dewetting (θ = 180) occurs. This expression follows from a balance of lateral forces acting on the three-phase contact line. However, it assumes that the surface does not undergo any structural changes in response to wetting, and neglects the vertical component of the liquid–vapor surface tension under the assumption that the solid is perfectly rigid. Neither of these assumptions is necessarily valid for polymer brushes. Brushes may swell in the presence of the solvent, changing their volume, composition and entropic elasticity. Moreover, polymeric materials can be unusually soft. As a result, the vertical force applied by the liquid–vapor interface may deform the surface, pulling it upward to form a “wetting ridge” near the three-phase contact line. In more extreme cases, the system may even deform on the length scale of the droplet, resulting in Neumann wetting. However, this has mostly been observed in extremely soft gels, rather than brushes.^192,193^

#### 3.4.1 Effects of Brush Parameters on Wetting State

One interesting aspect of wetting in polymer brushes is that only partial wetting is observed for many combinations of polymers and good solvents, when complete wetting might be expected based on classical arguments of solvation energy. Cohen Stuart et al. investigated this phenomenon through self-consistent field studies and experimental contact angle measurements, and propose an explanation based on the interaction between polymers and the liquid–air interface. Based on the surface activity of many water-soluble polymers, they suggest that the free end of polymer chains in the brush may adsorb at the liquid–air interface that is formed by a droplet on the surface despite being in a good solvent. Since releasing the adsorbed chain from the surface would increase the interfacial energy, this creates a (local or global) minimum in the free energy at a finite contact angle, stabilizing the partial wetting state.^84^

The wetting behavior of polymer melts on brushes forms a noteworthy example of the nonclassical wetting behavior of brushes. First, we discuss the case of chemically identical melts and brushes. Maas et al. studied this situation for a variety of grafting densities, grafted chain lengths and melt chain lengths using a scaling theory, self-consistent field calculations, and AFM imaging of a polystyrene melt/brush system.^194^ On a substrate that is partially wet by the polymer melt, a low density of grafted chains (below the critical density for brush formation) induces a transition to complete wetting. This is the result of a trade-off between the free energy of the grafted chains, which gain entropy and reduce their surface energy by interacting with the melt, and that of the melt polymers, which lose entropy as their movement and conformation are restricted by the presence of the surface. This complete wetting regime persists as grafting densities increase and the system becomes brush-like, as one might expect for a chemically identical surface and liquid. However, an upper grafting density exists at which the brush becomes too dense to accommodate the melt chains. At this point, the brush behaves approximately as an energetically neutral hard surface, which once again results in partial wetting by the melt,^195^ a phenomenon known as autophobicity. The loss of entropy at the brush–melt interface had previously been reported by Reiter and Khanna.^196^ Moreover, the lower and upper grafting density limits for complete wetting are expected to meet for very long melt chains, meaning that the melt will always partially wet the surface. However, this was not experimentally observed, which was attributed to metastability of the thick melt layer. In fact, a self-consistent field study by Matsen and Gardiner suggests that complete wetting may always be a metastable state.^197^ While this is a difficult claim to test, X-ray reflectivity results for a polystyrene melt/brush system by Zhang et al. show an approximate quantitative match with this self-consistent field theory.^198^

**Figure 15:**
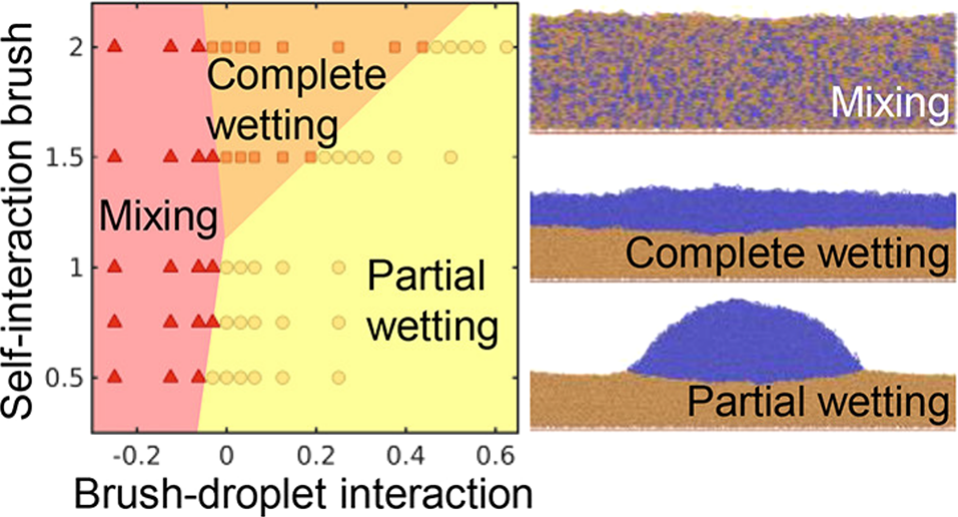
Phase diagram of wetting behavior for an oligomer droplet on a polymer brush as a function of the polymer self-attraction and the polymer-droplet interaction energy; the case of a chemically identical brush and drop is found on the vertical line at 0. Conventionally, mixing would be expected for all nonpositive interaction energies, but an additional attraction is required due to autophobicity effects. Reproduced with permission from ref (199). Copyright 2019 American Chemical Society.

Mensink et al. investigated the case of chemically distinct melts on brushes through coarse-grained molecular dynamics simulations.^199,200^ This resulted in a phase diagram distinguishing partial wetting, complete wetting, and mixing of the melt and brush as a function of the brush–melt interchange energy (a quantity linear with the Flory–Huggins parameter, as discussed in section 2.4) and the brush self-interaction strength. While the general shape of the phase diagram (shown in Figure 15) was well described by a classical enthalpic approach, the exact position of phase boundaries deviated due to the negative excess entropy at the brush–melt interface. Moreover, entropic contributions result in significant deviations of the contact angle from Young’s law in the partial wetting regime.^199^ Transitions between partial wetting, complete wetting and mixing are also strongly influenced by the chain length of the melt polymers, with shorter chains favoring complete wetting and mixing. This is explained by the fact that shorter chains gain substantially more translational entropy from the additional accessible volume than longer chains.^200^ Finally, for weak brush self-interactions, resulting in a mechanically soft brush, no transition to Neumann behavior was observed. This contrasts with similar simulations of polymeric drops on soft gels by Cao and Dobrynin, in which brush-drop contact angles decreased as the gel became softer and a Neumann regime was reached.^192^

#### 3.4.2 Switching the Wetting State

As discussed in previous sections on the different applications, switchable surface properties can be produced by various types of brush chemistry and architecture, which can be utilized to switch the wetting state for brushes as well. For example, thermal switching can be achieved by lower critical solution temperature polymers such as PNIPAm, which is hydrophilic at room temperature but transitions to a hydrophobic state around 32 °C.^201^ Sun et al. employed PNIPAm brushes on rough surfaces to enhance this effect and create switchability between a superhydrophilic and superhydrophobic state.^202^ Ionic strength and pH are another widely applicable switching mechanism. Fielding et al. demonstrated reversible wettability switching in brushes of several weak polybases, using protonation by HCl vapors as the switching mechanism. In their initial deprotonated state, these brushes are hydrophobic, and display correspondingly high water contact angles. Upon protonation by HCl, the brushes become charged and hydrophilic, displaying a moderately hydrophilic contact angle. Sun et al.^203^ synthesized brushes containing both poly(methacrylic acid) (PMAA) and basic PDMAEMA in random copolymer, block copolymer, and “V-shaped” polymer architectures, with the latter indicating a PMAA block and a PDMAEMA block anchored to the substrate at the same point by a surface-reactive block in the middle of the chain.^204^ After exposure to acidic or basic environments, resulting in protonation of the PDMAEMA block or deprotonation of the PMAA block respectively, these brushes displayed very low contact angles with aqueous solutions of the same pH as the switching solution. For approximately neutral pH, however, the copolymer is uncharged, and behaves hydrophobically. Demirci et al. produced polymer brushes of the ionic liquid 1-vinyl-3-butylimidazolium bromide functionalized with cyclodextrin, a common host group in supramolecular chemistry. Anion exchange, in which the bromide was replaced with the highly cyclodextrin-compatible bis(trifluoromethane)sulfonimide ion, resulted in a switch from hydrophilic to hydrophobic wetting behavior.^205^ This may be due to the tighter binding of counterions to the polymer chains via the host–guest interaction, weakening the ionic character of the brush.

**Figure 16:**
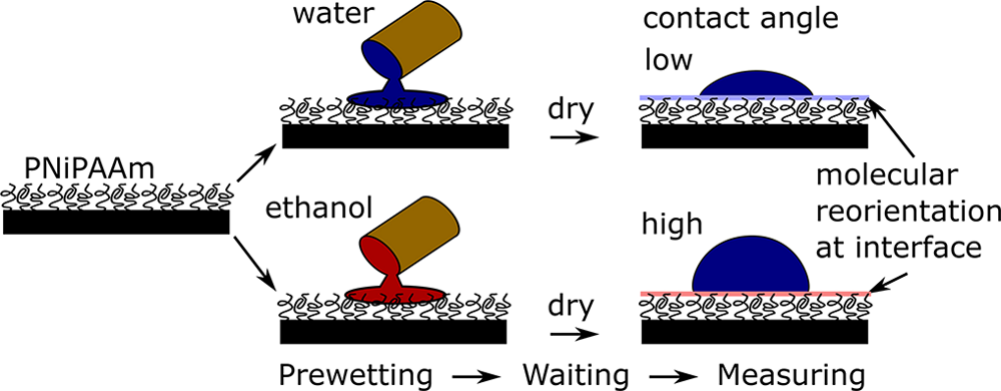
Prior wetting history of PNIPAm brushes has a persistent effect on the orientation of functional groups at the brush-air interface, resulting in differences in wetting behavior. Reproduced with permission from ref (206). Copyright 2021 Elsevier.

Schubotz et al. show not only switchability of the water contact angle on PNIPAm brushes by prior wetting with water or ethanol, but also observe a long-term memory effect.^206^ As qualitatively illustrated in Figure 16, prewetting with water results in a reduction of the water contact angle in the wetted area, whereas prewetting with ethanol increases the water contact angle, leading to a range of (advancing) contact angles from 25° to 65° on otherwise identical brush samples. This effect persisted after drying the brush and for periods of months. The memory effect is attributed to the rearrangement of PNIPAm at the surface in response to different solvent conditions, resulting in the exposure of either the amide side group or the alkane backbone. This rearrangement is confirmed by sum frequency generation spectroscopy.

#### 3.4.3 Antifogging and Anti-icing

**Figure 17:**
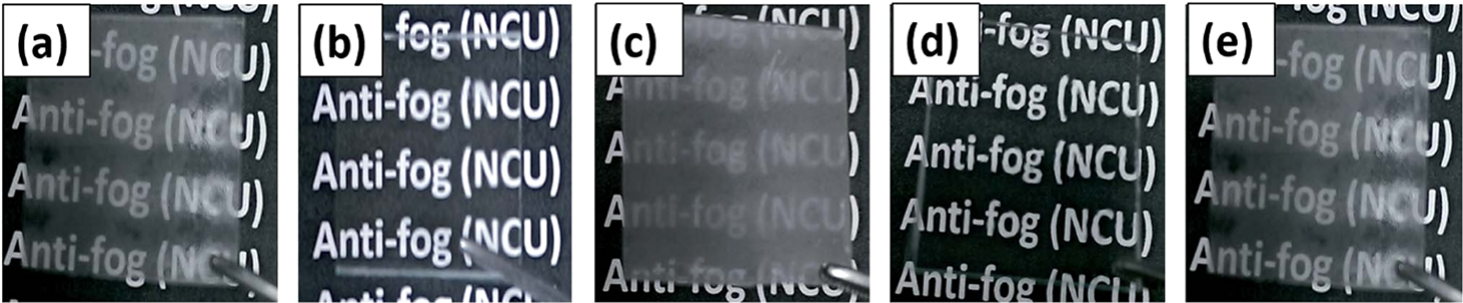
Antifogging properties of polyelectrolyte brushes: (a) untreated glass; (b) superhydrophilic (thin) PSBMA brush; (c) hydrophilic (thick) PSPBA brush; (d) superhydrophilic PSBVI brush; (e) hydrophilic PSBVI brush. Reproduced with permission from ref (207). Copyright 2016 Royal Society of Chemistry.

An everyday application of wetting control is found in antifogging and anti-icing surfaces. The formation of small water droplets or ice crystals on surfaces can be a problem when the surfaces in question are, e.g., glasses, windows, or optical instruments. By scattering incident light, such drops or crystals reduce the light transmission and visibility through a surface. Interestingly, both hydrophobic^208^ and hydrophilic^209^ surfaces are suitable for antifogging applications, since hydrophobic surfaces repel water altogether and strongly hydrophilic ones favor complete wetting, leading to the formation of a continuous liquid film. Howarter and Youngblood combined self-cleaning and antifogging properties on one surface using a brush of PEG chains capped with perfluorinated alkane segments. These brushes rearrange to present PEG segments at their surface in an aqueous or humid environment, and fluorinated segments when in contact with organics, tested in this study with hexadecane.^210^ Due to the low adhesion of hexadecane to the fluorinated surface and the hydrophilicity of PEG, the organic droplets could be removed from the surface by submersion in water. Ezzat and Huang investigated antifogging and anti-icing properties using poly(sulfobetaine methacrylate (PSBMA) and poly(sulfobetaine vinylimidazole) (PSBVI) brushes of different thicknesses.^207^ Thin brush layers of these polyzwitterions were found to be more hydrophilic than thicker ones, an effect attributed to self-association of the ionic groups in the brush^211^ and related to the anomalous swelling of sulfobetaine-functionalized polymers observed in ref (57) (see section 2.2). Both of the superhydrophilic thin brushes were found to display strong antifogging and anti-icing properties, with the antifogging effect shown in Figure 17. The anti-icing properties of the thinner brushes were attributed to their low surface roughness, providing minimal nucleation points for nucleation of ice growth.^207^ Antifogging and anti-icing have been observed in a variety of other polyelectrolyte and polyzwitterionic coatings due to their hydrophilic nature.^212,213^ It has also been suggested that bound water in the surface layer of brush coatings contributes to anti-icing by reducing the adhesion of ice on the brush surface.^214,215^

#### 3.4.4 Wetting Dynamics

The responsive character of brushes influences the dynamics of wetting as well. Shiomoto et al. studied wetting by water drops on a substrate patterned with hydrophilic PSPMA brush and hydrophobic fluoroalkylsilane monolayer stripes using optical microscopy, dying the water and the PSPMA brush for contrast. They found that this setup also facilitates visualization of the precursor film, the microscopically thin layer that spreads ahead of the macroscopic contact line when a liquid wets a surface.^216^ The spreading of the drop itself was found to follow a classical Tanner’s law time scaling to within reasonable corrections. However, the precursor film dynamics displayed two different regimes: the exact time scaling exponent depends on the liquid volume at the start of the experiment, but always transitions to an exponent of 0.6 for longer times. This was suggested to mark the transition from an adiabatic precursor film, where spreading is accelerated by the conversion of potential to kinetic energy in the flattening of the liquid drop, to a diffusive precursor film, which spreads purely under surface forces. However, the diffusive spreading exponent still differs from the classical value of 0.5 for general surfaces.^217^ The proposed mechanism underlying this is a combination of the large hydration energy of the brush driving wetting and energy dissipation by chain stretching slowing down the front. The precise origin of this scaling remains to be determined; however. Etha et al. used molecular dynamics to study a similar system, consisting of a brush wet by a drop of chemically identical oligomers. They identified a time scaling *r* ∼ *t*^1/4^ and an equilibrium drop radius of *r*_eq_ ∼ ρ_g_^–1/3^, consistent with a scaling approach in which the capillary driving force is balanced by viscoelastic forces resulting from the drop-substrate interaction.^218^ Moreover, they investigate the swelling dynamics of polymer chains as a function of grafting density via the brush height in the early stages of the wetting process, and find that the initial swelling response follows an approximate power law *h* ∼ *t*^δ^, where the exponent δ is typically smaller than unity and decreases as the grafting density of the brush increases, resulting in the intuitive conclusion that denser brushes display a slower swelling response. Thiele and Hartmann developed a model for the spreading of a drop on a polymer brush, based on gradient dynamics on a free energy expression accounting for capillary effects, brush wetting and brush elasticity.^219^ The dynamics are simplified to hydrodynamics within the drop, exchange of solvent between the brush and the drop, and diffusion within the brush, meaning that transport within the droplet and the brush are not coupled. The mesoscopic contact angles (Neumann angles at the approximate three-phase point) are found to evolve exponentially toward their equilibrium value, in accordance with other theoretical work, but show complex dynamics on short time scales as a result of the interplay between swelling, wetting and hydrodynamics.

#### 3.4.5 Outlook on Wetting Control

Since wetting is inherently tied to surface energy, fundamental results and experimental possibilities are closely linked in this context. Improving our understanding of the structure and width of the brush-air interface could provide additional, nonchemical parameters for tuning wetting behavior. Moreover, sorption kinetics in polymer brushes are not thoroughly explored. The various works on wetting dynamics we discuss all clearly illustrate the relevance of the brush swelling kinetics, suggesting a need for further research. This could also help in determining the relative importance of brush swelling, diffusion through the brush and hydrodynamics in wetting dynamics, which would enable predictions of wetting dynamics in experimental systems.

## 4 Conclusion

In this article, we have provided an overview of the state of the art with respect to polymer brushes in air, and we identified promising future avenues of research. On the fundamental side, many important results for brushes in liquid appear to extend to brushes under vapors as well. However, some open questions specific to the gas phase remain. The structure of the brush-air interface and the associated surface energy are a particularly relevant example, as, e.g., wetting behavior and mechanical properties of brush-functionalized surfaces will likely be dominated by the interfacial region of the brush. We anticipate that recently developed synthesis routes to fluorescently label brush polymers,^220,221^ can provide new insights on interfacial compositions for brushes in air. Additionally, predicting free volume within the brush is still a challenge. As illustrated by various results discussed in section 2.2 (for instance: refs (32 and 65)), free space within the brush alters the thermodynamics of vapor absorption and the brush response dramatically. This also touches on the topic of brush dynamics and kinetics: while the equilibrium behavior of polymer brushes is understood to a reasonable degree, effects such as solvent-induced glass transitions and vapor sorption kinetics have not been documented as thoroughly. Both of these topics are closely related to mobility and relaxation times of the brush, suggesting this as an avenue of further research. This is not only of great theoretical interest, as it relates to the ongoing research into polymeric glass transitions in general, but also of practical importance, since response times are at least as important as equilibrium behavior in switchability and sensing applications.

Despite the open fundamental questions, the works featured in the second half of this review show that useful and innovative applications of polymer brushes in air are already possible. Scaling these applications up beyond laboratory demonstrations remains a challenge, however. Novel techniques for applying polymer brush coatings are needed to make large-scale application feasible. Grafting-to methods remain somewhat restrictive with respect to brush architecture and grafting density, whereas the most common grafting-from strategies (surface-initiated radical polymerizations) usually require an oxygen-free environment. Additionally, both strategies only effectively utilize a small fraction of the monomer or polymer content of the reaction mixture. The use of, e.g., oxygen-consuming additives^222^ or filter paper-assisted surface-initiated Cu^0^-mediated polymerizations^213^ could make grafting-from in air possible, with the added benefit that the polymerization naturally terminates once the oxygen consumer is exhausted. This could allow for control of the approximate chain length via the composition of the reaction mixture. In related situations, brush coatings in practical settings will need to be stable under fluctuations in temperature and chemical environment and possibly under mechanical stresses, all of which may be large depending on the intended application. Many anchoring strategies do not yet meet this criterion, leading to degrafting^223−226^ even under relatively mild conditions in humid air.^61^ While this issue is relatively well-known and research on stable anchoring strategies is ongoing,^227−229^ further developments may be necessary to realize robust brush coatings.

Notwithstanding the remaining challenges, we are optimistic about the state of research on polymer brushes in air. First, the works cited in this review clearly show the potential utility of brush-based technologies in the gas phase. Additionally, many of the fundamental questions we raise are closely related to outstanding questions in broader polymer (brush) research. We see this as indicative of the maturing of brush-in-air research, and we are excited to see the further development of this subject.
